# Integrated Analysis of the microRNA–mRNA Network Predicts Potential Regulators of Atrial Fibrillation in Humans

**DOI:** 10.3390/cells11172629

**Published:** 2022-08-24

**Authors:** Rong Wang, Emre Bektik, Phraew Sakon, Xiaowei Wang, Shanying Huang, Xiangbin Meng, Mo Chen, Wenqiang Han, Jie Chen, Yanhong Wang, Jingquan Zhong

**Affiliations:** 1 The Key Laboratory of Cardiovascular Remodeling and Function Research, Chinese Ministry of Education, Chinese National Health Commission and Chinese Academy of Medical Sciences, The State and Shandong Province Joint Key Laboratory of Translational Cardiovascular Medicine, Department of Cardiology, Qilu Hospital, Cheeloo College of Medicine, Shandong University, 107 Wenhuaxi Road, Jinan 250012, China; 2 Department of Cardiology, Harvard Medical School, Boston Children’s Hospital, 300 Longwood Avenue, Boston, MA 02115, USA; 3 Department of Medicine, Cardiovascular Division, Harvard Medical School, Brigham and Women’s Hospital, Boston, MA 02115, USA; 4 Cancer and Blood Disorders Center, Boston Children’s Hospital, Dana-Farber Cancer Institute, Harvard Medical School, Boston, MA 02215, USA; 5 Department of Cardiac Surgery, Qilu Hospital, Cheeloo College of Medicine, Shandong University, 107 Wenhuaxi Road, Jinan 250012, China; 6 Department of Operating Room, Qilu Hospital, Cheeloo College of Medicine, Shandong University, 107 Wenhuaxi Road, Jinan 250012, China; 7 People’s Hospital of Dingtao District, A 056, Zhanqian Road, Dingtao District, Heze 274100, China; 8 Department of Anesthesia and Surgery, Provincial Hospital of the First Medical University of Shandong Province, No. 324 Jingwu Weiqi Road, Jinan 250021, China; 9 Department of Cardiology, Qilu Hospital (Qingdao), Cheeloo College of Medicine, Shandong University, 758 Hefei Road, Qingdao 266035, China

**Keywords:** heart disease, atrial fibrillation, atrial fibrosis, transcriptome, microRNA, RNA sequencing, SYNDECAN-1, miR-302

## Abstract

Atrial fibrillation (AF) is a form of sustained cardiac arrhythmia and microRNAs (miRs) play crucial roles in the pathophysiology of AF. To identify novel miR–mRNA pairs, we performed RNA-seq from atrial biopsies of persistent AF patients and non-AF patients with normal sinus rhythm (SR). Differentially expressed miRs (11 down and 9 up) and mRNAs (95 up and 82 down) were identified and hierarchically clustered in a heat map. Subsequently, GO, KEGG, and GSEA analyses were run to identify deregulated pathways. Then, miR targets were predicted in the miRDB database, and a regulatory network of negatively correlated miR–mRNA pairs was constructed using Cytoscape. To select potential candidate genes from GSEA analysis, the top-50 enriched genes in GSEA were overlaid with predicted targets of differentially deregulated miRs. Further, the protein–protein interaction (PPI) network of enriched genes in GSEA was constructed, and subsequently, GO and canonical pathway analyses were run for genes in the PPI network. Our analyses showed that TNF-α, p53, EMT, and SYDECAN1 signaling were among the highly affected pathways in AF samples. SDC-1 (SYNDECAN-1) was the top-enriched gene in p53, EMT, and SYDECAN1 signaling. Consistently, SDC-1 mRNA and protein levels were significantly higher in atrial samples of AF patients. Among negatively correlated miRs, miR-302b-3p was experimentally validated to suppress SDC-1 transcript levels. Overall, our results suggested that the miR-302b-3p/SDC-1 axis may be involved in the pathogenesis of AF.

## 1. Introduction

Atrial fibrillation (AF) is the most common form of cardiac arrhythmia in humans and is often characterized by rapid and irregular beating of the atria [1,2]. AF affected ~33.5 million people worldwide in 2010, with this number increasing by ~5 million new patients each year [3,4]. The prevalence of AF progressively increases in an aging society, leading to reduced life quality and/or elevated mortalities [5,6]. It was reported that an average of 130,000 AF patients die in the USA [7] and that AF affects 2–3% of the European population [4,8,9]. Additionally, AF is estimated to affect ~72 million people in Asia, and the number of patients with AF-related strokes is estimated to reach 2.9 million in the next 30 years [3,10].

AF often starts with a trigger in a vulnerable substrate that leads to short episodes of rapid ectopic firing and re-entry of electrical signal in the atria. Over time, AF may turn into a permanent condition through the atrial remodeling process, which is central to AF [11]. Structural remodeling in the atria is often identified by atrial fibrosis, dilation, and abnormal conduction of electrical pulses, while electrical remodeling results in abnormal impulse generation due to ion channel dysfunction [12,13]. Current therapeutic approaches have limited efficacy and adverse effects in the treatment of AF due to a lack of molecular understanding of AF substrates and mechanisms [13,14]. Therefore, there is an urgent need to expand mechanistic knowledge on AF development and progression.

Recent developments in AF research identified microRNAs (miRs) as novel regulators in AF [15,16]. MiRs are classified as a subtype of short non-coding RNAs with a typical length of 18–24 nucleotides, and can negatively regulate protein expression levels of their target genes through binding with the 3’ UTR of mRNAs [17] with the exception that some may directly interact with and regulate the function of proteins, e.g., ion channels [18,19]. After the initial discovery of miR-1 function in heart development, a large number of research efforts focused on investigating miRs and their function in the cardiovascular system. Indeed, a large number of miRs is abnormally expressed in various cardiac disorders [20,21,22]. Gene expression of patient samples with AF history and other functional studies identified miRs in deregulation of ion channels, leading to electrical remodeling [15,16,17]. Structural remodeling through cardiac fibrosis was also shown to be regulated by miRs [18,19]. However, current knowledge of miR function in AF is limited and requires more research. Thus, analysis of miR and target gene expression networks can improve our knowledge of regulatory mechanisms of miRs in AF induction and progression [20,21].

**Figure 1:**
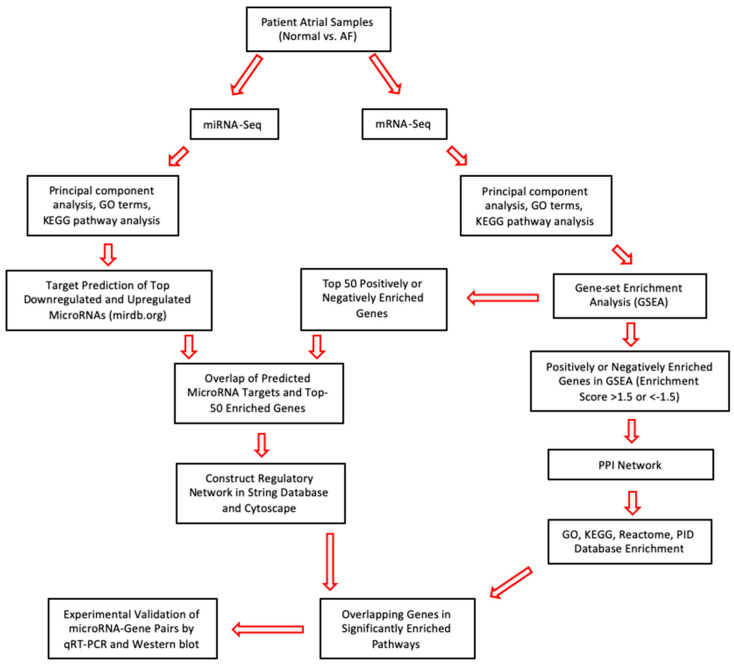
Flowchart of RNA-Seq Experimental and Target Prediction Strategy.

In this study, we aimed to predict novel miR–mRNA pairs that may play roles in the pathogenesis of AF. Thus, we performed RNA sequencing of miRs and mRNAs from atrial samples of patients with a history of persistent AF and no AF with normal sinus rhythm (SR) (Table 1; Figure 1). Several analyses including gene ontology (GO), Kyoto encyclopedia of genes and genomes (KEGG) pathway, and gene-set enrichment analyses (GSEA) were run for differentially expressed genes (DEGs) and top-enriched genes in GSEA of DEGs. Then, we profiled the interaction network of negatively correlated miR–mRNA pairs using Cytoscape. Our results revealed 15 gene and 13 miR candidates, among which gene expression levels of miR-302b-3p and SDC-1 were experimentally confirmed to be negatively correlated in atrial tissue samples. Further, overexpression of miR-302b-3p by mimics significantly reduced SDC-1 levels in vitro. SDC-1 is known for its function in the cardiac fibrosis [23,24], and our analyses found SDC-1 in close interaction with AF-associated genes, e.g., SELE. Briefly, our results suggest that the miR-302b-3p/SDC-1 axis may function in AF via modulating atrial fibrosis.

## 2. Results

### 2.1. Analysis of Differentially Expressed miRs in Persistent AF

**Figure 2:**
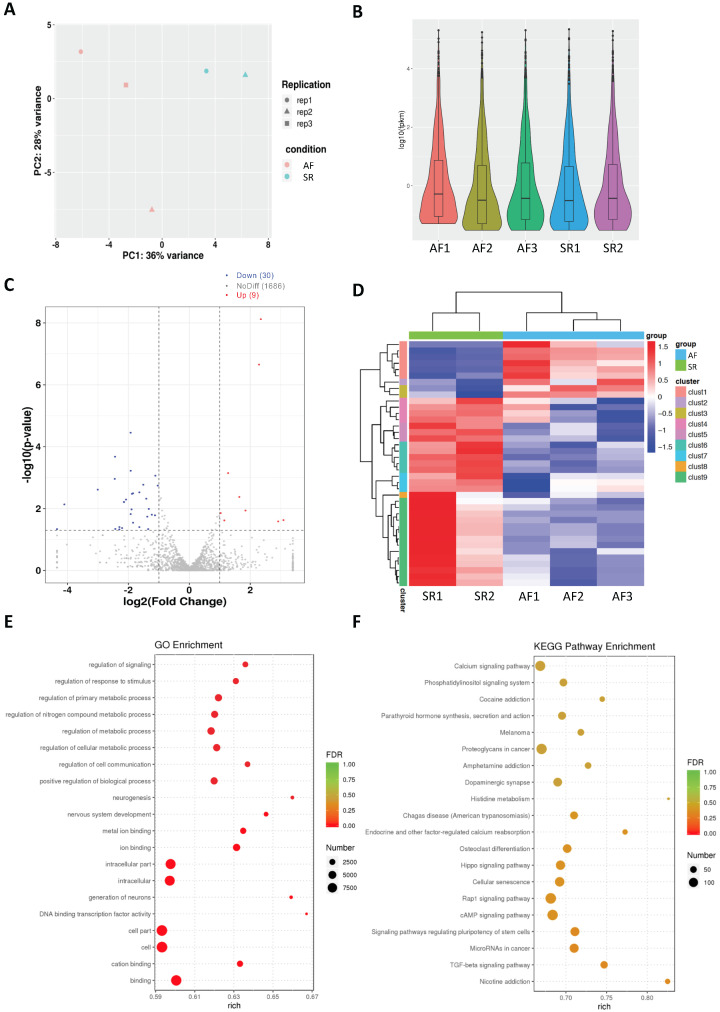
miR-Seq analysis for DEGs in the SR control and AF groups. (**A**) PCA analysis of SR and AF patient samples. (**B**) The average number of miRs analyzed from patients and the ctrl group in miR-seq. (**C**) Volcano plot shows significantly upregulated (red), downregulated (blue), and non-significant miRs (grey). Significant miRs are selected based on |log_2_FoldChange| > 1 and *p* < 0.05. (**D**) Heat map matrix shows clustering of differentially expressed miRs between the SR and AF groups. (**E**) GO and (**F**) KEGG pathway enrichment analysis of top deregulated pathways.

Our miR-seq of the SR control and AF groups detected total raw read counts of >42 × 10^6^ miRs and clean read counts of >33 × 10^6^ miRs with a cut-off sequence length of ≥18 nucleotides. Principal component analysis (PCA) of samples from AF or SR patients showed that each group was prominently clustered for miR-seq (Figure 2A). miR expression distribution in each sample was shown in terms of log_10_(fpkm) (Figure 2B). A total of 1725 miRs were detected in our miR-seq. Differentially expressed miRs with a threshold of |log_2_(FoldChange)| > 1 and a statistical significance of *p* < 0.05 were identified (Figure 2C), and significantly differentially expressed miRs were illustrated in a heatmap (Figure 2D). There were 9 upregulated and 30 downregulated miRs (Figure 2A–D; Table 2). GO and KEGG pathway analysis found that various pathways were affected in AF (Figure 2E,F). TGF-β was among the highly upregulated ones in miR-seq (Figure 2E).

### 2.2. Analysis of Differentially Expressed mRNAs in Persistent AF

**Figure 3:**
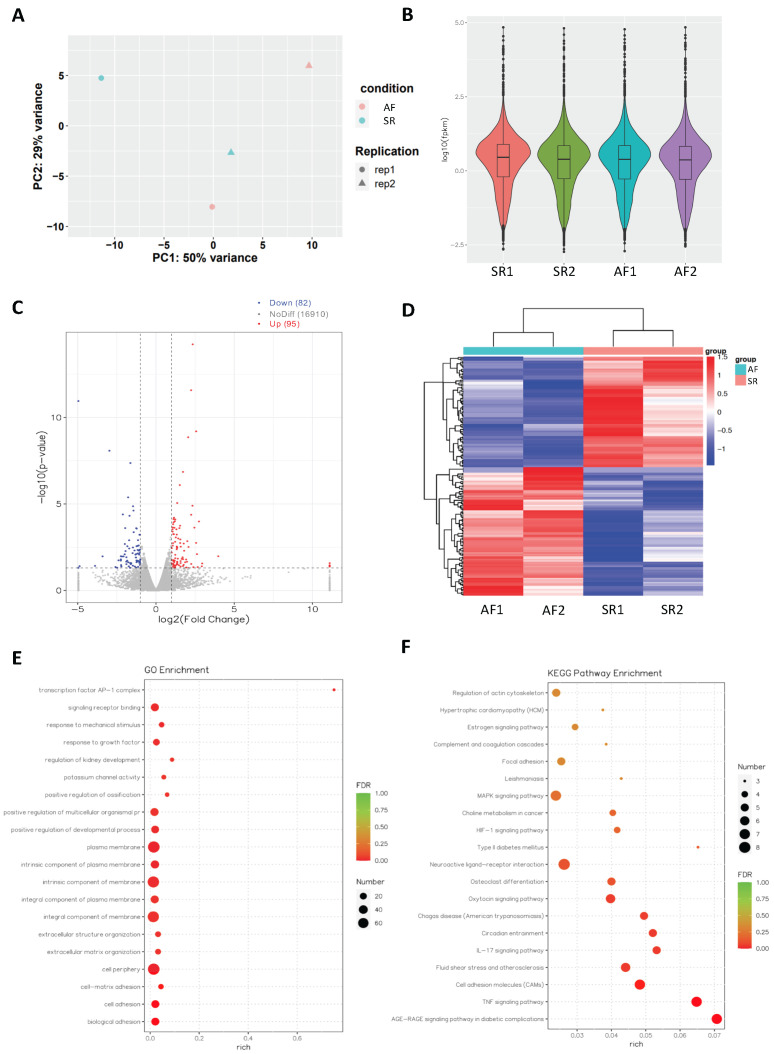
mRNA-seq analysis for DEGs in the SR control and AF groups. (**A**) PCA analysis of SR and AF patient samples. (**B**) The average number of genes analyzed from patients and the ctrl group in mRNA-seq. (**C**) Volcano plot shows significantly upregulated (red), downregulated (blue), and non-significant (grey) genes. Significant genes are selected based on |log_2_(FoldChange)| > 1 and *p* < 0.05. (**D**) Heat map shows clustering of differentially expressed genes between the SR and AF groups. (**E**) GO and (**F**) KEGG pathway enrichment analysis of top deregulated pathways.

More than 49 × 10^6^ raw read counts of mRNAs were detected in the SR and AF groups. PCA of samples from AF or SR patients showed that each group was prominently clustered (Figure 3A). mRNA expression distribution in each sample was shown in terms of log_10_(fpkm) (Figure 3B). A total of 17,087 genes were detected in mRNA-seq. Differentially expressed genes (DEGs) with a threshold of |log_2_(FoldChange)| > 1 and a statistical significance of *p* < 0.05 were identified (Figure 3C), and significantly differentially expressed genes were illustrated in a heatmap (Figure 3D). There were 95 upregulated and 82 downregulated mRNAs (Figure 3A–D). GO and KEGG pathway analysis found that various pathways were affected in AF (Figure 3E,F). mRNA-seq found cell adhesion molecules and the TNF-α signaling pathway among the highly enriched pathways (Figure 3E).

### 2.3. Network Analysis Downregulated miRs and Upregulated Genes and Selection of Candidate Pairs

**Figure 4:**
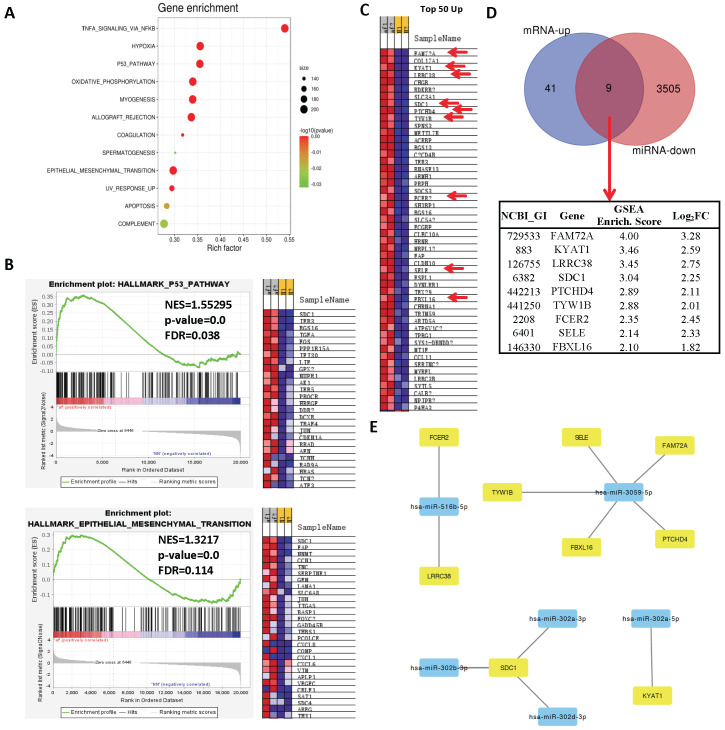
Analysis of upregulated genes and selection of candidate miR–mRNA pairs. (**A**) Gene-set enrichment analysis (GSEA) of upregulated genes. (**B**) Enrichment plots of some of the top enriched pathways. (**C**) Heat map showing top 50 positively enriched genes in GSEA based on enrichment score. (**D**) Venn diagram showing overlapping genes between top 50 positively enriched genes in GSEA (blue) and all predicted targets of top 11 downregulated miRs in RNA-seq (red). A total of 9 overlapping genes between groups were selected as potential candidates and their enrichment scores and log_2_(FoldChanges) are listed in the table. (**E**) Interaction network of positively enriched genes in GSEA and their negatively correlated microRNAs.

To determine affected gene sets among differentially upregulated genes, we ran a gene-set enrichment analysis (GSEA) and showed that 12 pathways were significantly enriched with differentially upregulated genes (Figure 4A). Among these pathways, TNF-α inflammatory signaling was the top affected pathway, which is usually upregulated in AF and is a sign of the ongoing inflammatory process [25]. Additionally, other hallmark pathways, including p53 signaling and epithelial-to-mesenchymal transition (EMT), were highly enriched (Figure 4A,B). To determine miR–gene pairs, we searched predicted targets of top 11 differentially downregulated miRs (Table 2) in the miRDB database (www.mirdb.org (accessed on 7 May 2021)) and constructed an interaction network with negatively correlated genes in RNA-seq by using Cytoscape 3.8.2 [26]. To prioritize genes with relatively high enrichment in RNA-seq, we included only top 50 enriched genes from GSEA (Figure 4C) and overlaid them with predicted targets of top 11 differentially downregulated miRs, which resulted with 9 genes (FAM72A, KYAT1, LRRC38, SDC1, PTCHD4, TYW1B, FCER2, SELE, and FBXL16) (Figure 4D), 6 miRs (miR-3059-5p, miR-302a-5p, miR-516b-5p, miR-302b-3p, miR-302d-3p, and miR-302a-3p), and 11 interactions between them (Figure 4E; Table 3). GO terms of these nine identified genes showed no shared functional annotation (www.david.ncifcrf.org (accessed on 12 May 2021)), except six of them (LRRC38, SDC-1, PTCHD4, TYW1B, FCER2, and SELE) being plasma membrane-associated proteins (data not shown). Overall, these highly enriched genes and their corresponding miRs are potential candidates that may involve in AF pathogenesis.

### 2.4. Network Analysis of Upregulated miRs and Downregulated Genes and Selection of Candidate Pairs

**Figure 5:**
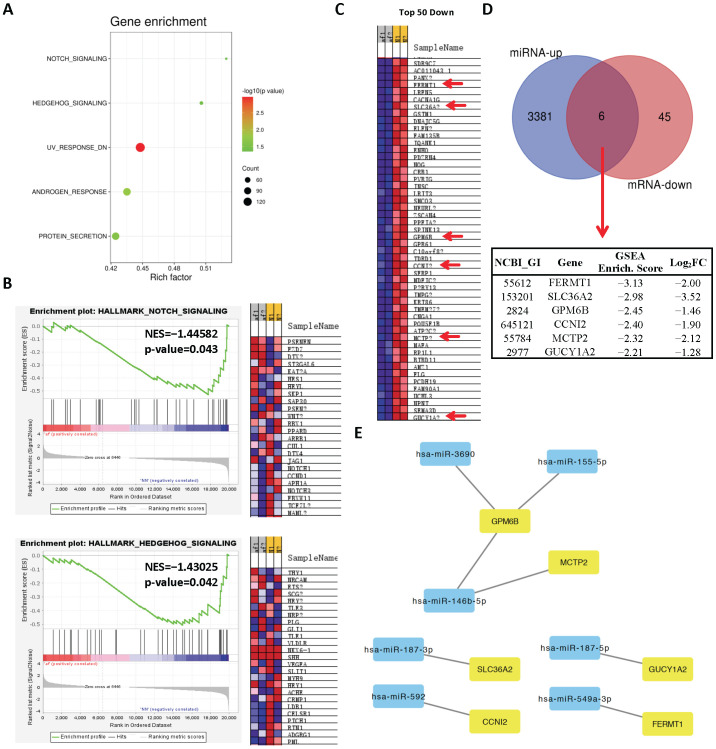
Analysis of downregulated genes and selection of candidate miR–mRNA pairs. (**A**) Gene-set enrichment analysis (GSEA) of downregulated genes. (**B**) Enrichment plots of some of the top enriched pathways. (**C**) Heat map showing top 50 negatively enriched genes in GSEA based on enrichment score. (**D**) Venn diagram showing overlapping genes between top 50 negatively enriched genes in GSEA (blue) and all predicted targets of top 9 upregulated miRs in the RNA-seq dataset (red). A total of six overlapping genes between groups were selected as potential candidates and their enrichment scores and log_2_(FoldChanges) are listed in the table. (**E**) Interaction network of negatively enriched genes in GSEA and their negatively correlated miRs.

Our gene-set enrichment analysis (GSEA) for downregulated genes showed that five pathways were enriched among significantly downregulated genes (Figure 5A). Among these pathways, Notch and Hedgehog signaling were the top affected pathways (Figure 5B). To determine potential miR–gene pairs, we overlaid predicted targets of top 9 differentially upregulated miRs in Table 1 (www.mirdb.org (accessed on 7 May 2021)) and top 50 negatively enriched genes in GSEA (Figure 5C). A total of six common genes were identified as potential candidates (Figure 5D). Subsequently, we constructed an interaction network of negatively correlated pairs in Cytoscape 3.8.2 (Figure 5E). Our analysis identified six genes (FERMT1, SLC36A2, GPM6B, CCNI2, MCTP2, GUCY1A2), seven miRs (miR-146b-5p, miR-155-5p, miR-3690, miR-187-5p, miR-187-3p, miR-592, and miR-549a-3p), and eight interactions between them (Figure 5E; Table 4). Overall, these highly enriched genes and their corresponding miRs may involve in the pathogenesis of AF.

### 2.5. Selecting a Candidate Gene and Experimentally Validating Its Expression

We further analyzed genes in GSEA analysis to determine what pathways were highly affected by top-enriched genes in GSEA. The protein–protein interaction (PPI) network was constructed for the genes with an enrichment score of ES > 1.5 (Figure S1A) or ES < −1.5 (Figure S2A). Genes with at least one interaction and a confidence score >50 were included in the analysis. The PPI network of positively enriched genes showed that SDC-1 and SELE, some of the candidates in Figure 4E, were in direct or indirect interactions with a group of AF-associated genes (Figure S2A,B). p53 and TNF-α hallmark pathways were highly enriched by the genes in the PPI network (Figure 4B and Figure S2C). Additionally, canonical pathway analysis (www.gsea-msigdb.org (accessed on 21 March 2022)) showed significant enrichment of ATF2, AP-1, FRA, and SYNDECAN-1 pathways (Figure S2D). Indeed, SDC-1 was a shared gene in both p53 and EMT signaling (Figure 4B and Figure S2C) as well as in the SYNDECAN-1 pathway (Figure S2D), and thus stands out as a promising candidate possibly involving in structural remodeling of atria. Further, GO terms found SDC-1 involved in top significant terms (Figure S2E–G). As for negatively enriched genes, we found no significant interaction with AF-associated genes (Figure S3A,B). However, GPM6B, among the candidate genes in Figure 5E, was in a major interaction network with neuronal genes (Figure S3A,B). GPM6B was not found in any significantly enriched hallmark pathways (Figure S3C,D), despite GPM6B being a neuron-specific gene. However, neuron-related gene sets were highly enriched among genes from the PPI network. Additionally, neuron-related GO terms were consistently enriched and showed a strong association with GPM6B (Figure S3E). Thus, GPM6B was a considerably good candidate possibly involving in neuronal remodeling of atria.

**Figure 6:**
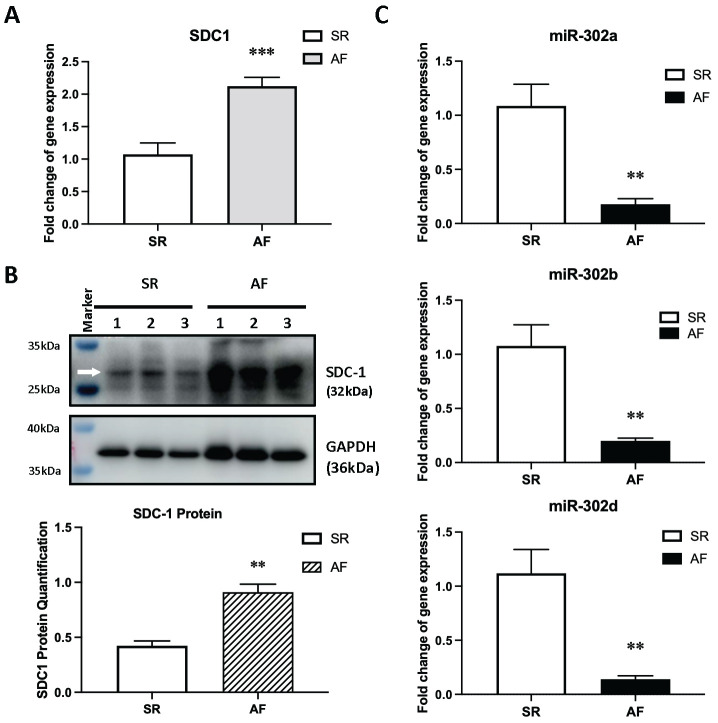
Validating expression of miR-302b-3p and SDC-1 in human atrial tissue. (**A**,**B**) Expression of SDC-1 (**A**) mRNA (*n* = 6) and (**B**) protein (*n* = 3) in SR (sinus rhythm) vs. AF atrial tissue samples. (**C**) Expression levels of miR-302 family in atrial tissue samples of the SR vs. AF groups (*n* = 6). ** *p* < 0.01 or *** *p* < 0.005.

As SDC-1 was in interaction with AF-associated genes (Figure S2A) and highly enriched in p53, EMT, and SYNDECAN-1 signaling pathways, we decided to experimentally validate SDC-1 and its corresponding miRs. MiR-302 family had a very high prediction score (96 out of 100) targeting SDC-1 gene (Table 3). We initially checked gene and protein expression levels of SDC-1 in human atrial tissue samples. AF patients had significantly elevated levels of SDC-1 mRNA (2.12 ± 0.13, *n* = 6 vs. 1.074 ± 0.18, *n* = 6 in ctrl) (Figure 6A) and protein expression (0.91 ± 0.07, *n* = 3 vs. 0.42 ± 0.04, *n* = 3 in ctrl) (Figure 6B). Consistently, expression levels of miR-302a-3p (0.18 ± 0.05, *n* = 6 vs. 1.09 ± 0.20, *n* = 6 in ctrl), miR-302b-3p (0.20 ± 0.03, *n* = 6 vs. 1.08 ± 0.20, *n* = 6 in ctrl), and miR-302d-3p (0.14 ± 0.03, *n* = 6 vs. 1.12 ± 0.22, *n* = 6 in ctrl) were significantly reduced in AF tissues (Figure 6C), showing a negative correlation with SDC-1.

### 2.6. MicroRNA-302b-3p Regulates SDC1 Expression In Vitro and May Involve in Atrial Fibrosis

**Figure 7:**
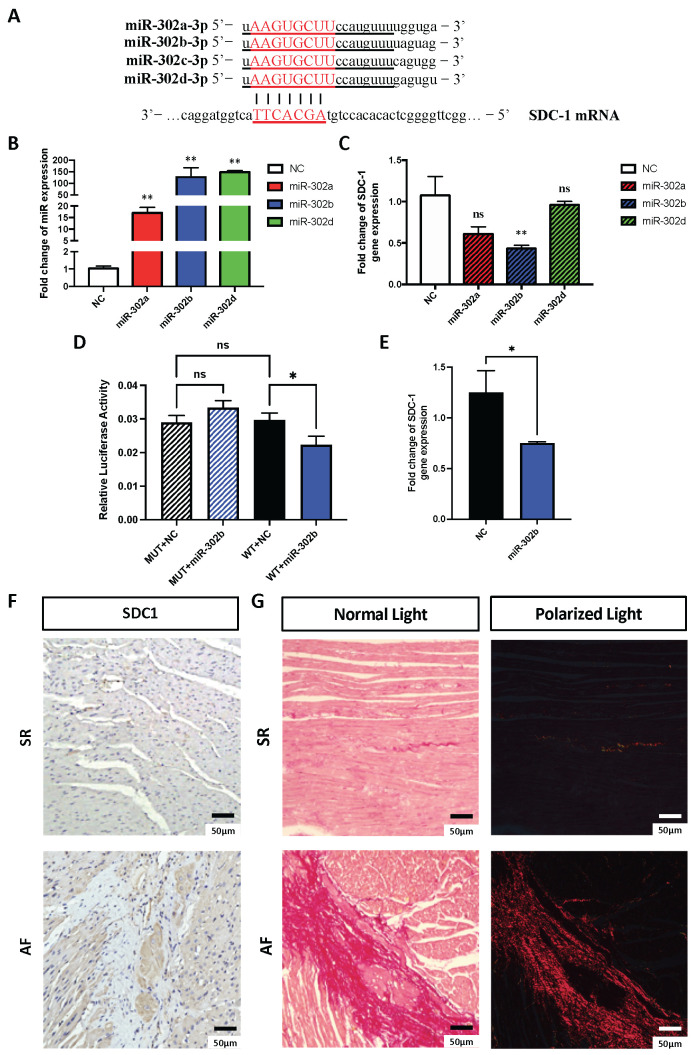
miR-302-3p may regulate atrial fibrosis by targeting the expression of SDC-1. (**A**) Conserved sequences of miR-302 family. Underlined sequences indicate conserved nucleotides including their seed sequences. Seed sequences were highlighted in red capital letters. (**B**) Transfection of negative ctrl (NC) (*n* = 4) vs. mimics of miR-302a-3p (*n* = 6), miR-302b-3p (*n* = 6), or miR-302d-3p (*n* = 4) into 293T cells. (**C**) qRT-PCR shows SDC-1 gene expression levels after transfection with NC (*n* = 6) or miR mimics (*n* = 4). (**D**) Luciferase assay for the mutant and wild-type 3’UTR of SDC-1 following transfection with control or miR-302b-3p mimics (*n* = 3). (**E**) Reduced SDC-1 levels in human dermal fibroblasts (HDF) transfected with miR-302b-3p mimics (*n* = 4) (**F**) Immunohistochemistry of SDC-1 in atrial tissues samples from SR (sinus rhythm) control and AF patients (**G**) Picrosirius red staining of collagen fibers in SR ctrl and AF patients. Total collagen appears in dark pink color (left column) with standard light microscopy. Collagen-I appears in red (right panel) with polarized light microscopy. * *p* < 0.05 or ** *p* < 0.01.

The miR-302-3p family has a conserved seed sequence and thus has a high overlap of their target genes (Figure 7A). To evaluate whether they all target SDC-1 and reduce mRNA levels, we transfected 293T cells with either of all three isotypes, which were found among top significantly downregulated miRs in our miRseq (Table 2), and showed efficient overexpression levels (miR-302a: 17.03 ± 2.36, *n* = 6; miR-302b: 127.1 ± 40.3, *n* = 6; miR-302d: 148.8 ± 5.81, *n* = 4; vs. ctrl: 1.03 ± 0.15, *n* = 4) (Figure 7B). However, only miR-302b-3p showed a significant reduction in SDC-1 mRNA levels (0.43 ± 0.04, *n* = 6 vs. 1.08 ± 0.23, *n* = 4 in ctrl) (Figure 7C). To validate this hypothesis, we performed a luciferase assay using wild-type and mutated nucleotide sequences of putative binding sites of miR-302b-3p on the 3’UTR of SDC1. Results showed a significant reduction in luciferase signal in 293T cells transfected with miR-302b-3p (0.022 ± 0.001, *n* = 3) compared to control-transfected cells (0.03 ± 0.001, *n* = 3), proving the direct interaction of SDC-1 with miR-302b-3p (Figure 7D). Moreover, transfection of human dermal fibroblasts (HDFs) with miR-302b-3p mimics consistently reduced SDC-1 gene expression in vitro (0.75 ± 0.01, *n* = 4 vs. 1.25 ± 0.22, *n* = 4 in ctrl) (Figure 7E). Finally, we found that SDC-1 levels were visibly increased in atrial tissue samples of AF patients compared to the SR control group (Figure 7F), and this was positively correlated with increased fibrosis, marked by elevation of the collagen matrix, in AF samples (Figure 7G). Overall, these all together suggested that the miR-302b-3p/SDC-1 axis may have a role in AF pathogenesis through the regulation of atrial fibrosis.

## 3. Discussion

Non-coding RNAs play critical roles in the development and pathogenesis of AF. Recent studies have predicted novel genes and non-coding RNAs by RNA-seq of the transcriptome in patient-derived atrial samples [27,28,29,30,31]. Here, in our RNA-seq data, we performed similar analysis techniques to profile differential expression of miRs (9 up and 30 down) and mRNAs (95 up and 82 down). To be able to identify potential miR–mRNA pairs, we followed a different strategy by further analyzing enriched genes in GSEA, which are highly enriched among all sequenced mRNAs, and overlaying them with differentially expressed miRs. Briefly, top 50 positively or negatively enriched genes (Figure 4D and Figure 5D, respectively) were overlaid with predicted targets of top differentially expressed miRs (Figure 4E and Figure 5E). An interaction network of common genes between two groups and their corresponding miRs was constructed by Cytoscape. Eventually, we identified 15 genes and 11 miRs in total that are negatively correlated in expression (Table 3 and Table 4), which will be discussed below in detail.

We linked nine positively enriched genes (Figure 4D) and six negatively regulated miRs (Figure 4E; Table 2). Among these genes, TYW1B, FCER2, SELE, and SDC-1 had some degree of connection with cardiovascular diseases including AF. TYW1B is associated with triglyceride metabolism in heart disease [32,33]. GWAS studies found novel variants of genes including TYW1B that are associated with altered triglyceride levels in heart disease. FCER2, a B-cell specific antigen involved in the inflammatory response, is associated with cardiovascular disease [34]. SELE (Selectin E) is an endothelial cell-specific surface protein involved in leukocyte attachment to endothelial cells. Previously, genetic variants of SELE were strongly associated with cardiovascular disease [35,36,37,38]. Interestingly, increased levels of soluble endothelial markers, including SELE, and endothelial dysfunction have been co-observed in AF patients [39,40]. Lastly, SDC-1 (SYNDECAN-1) is a transmembrane heparan sulfate proteoglycan, which plays a critical role in cell–cell adhesion and intercellular communication [41,42]. SDC-1 has previously not been associated with AF although it has been studied in various disease settings including heart disease [43,44,45,46,47,48]. On the contrary, FAM72A, KYAT1, LRRC38, and PTCHD4 had little or no known associations with cardiovascular diseases so far although they are moderately expressed in the heart and may have unidentified functions in AF. We also linked six negatively enriched genes (Figure 5D) and seven upregulated miRs (Figure 5E; Table 3). Among these, expression of SLC36A2 in epicardial adipose tissue is linked with an increased risk of coronary artery disease [49]. GPM6B regulates smooth muscle cell differentiation by controlling the TGF-β/Smad2/3 axis via direct interaction with TβRI (TGF-β receptor 1) [50]. Indeed, fibrosis, among the hallmarks of AF, is widely induced by TGF-β signaling, and smooth muscle abnormalities were coexistent with fibrosis in AF patients [51]. MCTP2 is an essential gene required for the proper development of the left ventricular outflow tract, and genetic mutations in this gene result in abnormal development of outflow tracts [52,53,54]. Additionally, genetic variants of MCTP2 have been associated with heart rhythm increase [55,56]. Lastly, a GUCY1A2 single-nucleotide polymorphism was associated with hypertension and is useful in the detection of patients predisposed to hypertension [57]. On the contrary, FERMT1 and CCNI2 have no direct link with any form of cardiovascular disease so far although they may have some unknown functions in AF. Altogether, given that some of these genes are already associated with cardiovascular diseases, they may involve in the pathogenesis of AF along with their corresponding miRs. Among these, SDC-1 stands out as a strong candidate based on our analyses, and its potential involvement in heart disease and AF will be extensively discussed below.

As for identified miRs, we found novel downregulated miRs (miR-302a-3p, miR-302a-5p miR-302b-3p, miR-302b-3p, miR-3059-5p, miR-516b-5p) which may regulate multiple top enriched genes (Table 3). Interestingly, several members of miR-302 were among the highly downregulated miRs, miR-302b-3p being the top significantly downregulated miR (Table 2). Among the upregulated miRs, miR-146b-5p was the top significantly upregulated miRs, which has been previously studied in the atrial fibrosis [58]. Additionally, some novel upregulated miRs were identified in our study including miR-549a-3p, miR-187-3p, miR-187-5p, miR-155-5p, miR-3690, and miR-592, which may have implications in AF through regulating their predicted target genes (Table 4). Among the selected miR–gene pairs, we experimentally validated that only FAM72A and SDC-1 were statistically changed in AF tissue samples (Figure 6 and Figure S1). As the prediction score of FAM72A/miR-3059-5p was not high enough (Table 3), we proceeded with investigating the potential interaction between SDC-1 and miR-302-3p.

**Figure 8:**
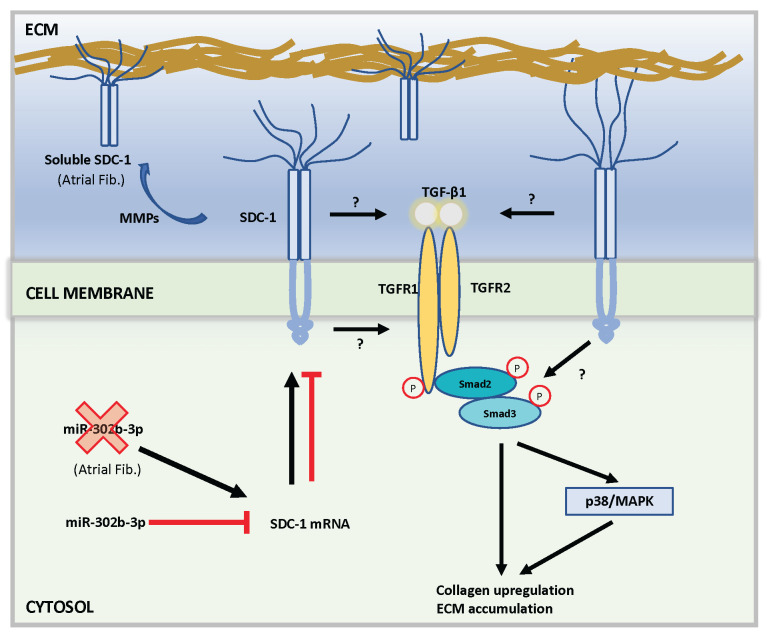
Proposed mechanism of SDC-1 in fibrotic remodeling of AF. Decreased levels of the miR-302-3p family will result in increased expression of SYNDECAN-1 in atrial cardiac fibroblasts, which will stimulate TGF-β/Smad2-mediated atrial fibrosis.

SDC-1 was reported to function in heart failure by promoting fibrosis through TGF-β/Smad2 [23,59,60] or p38/MAPK [24] pathway. Indeed, TGF-β was among the highly enriched signaling pathways in KEGG pathway analysis (Figure 2F). Additionally, our PPI network showed indirect interaction of SDC-1 with TGFA and a group of AF-associated genes (Figure S1A,B). Likewise, we found a prominent increase in SDC-1 protein expression (Figure 6B and Figure 7F) and collagen deposition in atrial tissue samples of AF patients (Figure 7G), suggesting that SDC-1 may involve in fibrosis in AF. On the other hand, cell adhesion molecules, including SDC-1, were highly enriched in the KEGG pathway analysis of DEGs (Figure 3F). Consistently, the PPI network of positively enriched genes in GSEA found the SYNDECAN-1 pathway as among the top enriched pathways (Figure S1D). Further, our data found that SDC-1 was the top enriched gene in both p53 and EMT hallmark pathways, which are among the top 10 significantly enriched pathways in GSEA of DEGs (Figure 4A) and GSEA of the PPI network (Figure S1C). Both p53 signaling [61,62] and EMT [63,64] are involved in various aspects of heart disease, including AF. More specifically, p53 signaling was shown to be linked with the premature senescence of atrial fibroblasts in AF, which results in a progressive increase in ECM accumulation and perivascular fibrosis [65,66] and is commonly observed in aging individuals [67]. On the other hand, it has been known that EMT is regulated by TGF-β in atrial fibroblasts and its effect is further strengthened under proinflammatory TNF-α signaling in AF [68]. To ensure a negative correlation between SDC-1 and miR-302 family in vitro, we checked gene expression of SDC-1 and miRs in atrial tissue samples and validated our RNA-seq results (Figure 6A–C). To validate the targeting of SDC-1 by its potential regulator, the miR-302-3p family, we transfected 293T cells with mimics of either of three isotypes, which are all significantly downregulated in our RNA-seq (Table 2), and validated that miR-302b-3p regulates SDC-1 expression in vitro (Figure 7A–E). Indeed, some members of the miR-302 family have been previously shown to target SDC-1 transcripts [69,70], supporting our findings. Collectively, the miR-302b-3p/SDC-1 axis may involve in AF through regulating atrial fibrosis, which is connected with profibrotic signaling by TGF-β, p53, and EMT (Figure 8).

Although pathological remodeling of non-cardiomyocytes alone can induce arrhythmia responses in humans [71] and SDC-1 is highly expressed in cardiac fibroblasts [23,59,72], it needs validation to prove whether SDC-1 function in AF is mediated through fibroblasts. Therefore, further studies on the cellular distribution of SDC-1 in atrial heart tissue may shed light on its mechanism in AF. As both the miR-302 family and SDC-1 have conserved sequences and function between humans and mice, mouse models may help elucidate molecular mechanisms of the miR-302b-3p/SDC-1 axis in the AF pathogenesis. Indeed, disease models with iPSC-derived fibroblasts [73] or CMs [74], which are physiologically more similar to native human cells, also offer a reliable platform for investigation of AF mechanisms as the tissue resources from patients are limited for extensive mechanistic research [74,75]. Experimental validations of miR-302b-3p/SDC-1 function and mechanism may help generate therapeutic approaches targeting this axis in the treatment of AF patients.

Although our study has methodological similarities with previously published works, we differently performed RNA-seq of mRNA and miRs from matched samples of all persistent AF patients. Persistent AF represents a more progressed stage of AF toward permanent AF. Here, we identified miR and gene pairs that have not been associated with AF in past studies, such as the interaction of miR-302 family miRs and transmembrane protein SDC-1.

Our study had some limitations. We excluded the second female AF sample from mRNA-seq analysis as it did not pass quality control for mRNA-seq. Additionally, some important genes or miRs might have been missed out due to our small sample size. Regardless of this issue, we had sufficient miR (> 42 × 10^6^) and mRNA (49 × 10^6^) reads detected in our RNA-seq data, and could successfully detect differential expression of some of the previously identified miRs and genes. For example, miR-146b-5p—the most significantly upregulated miR in our miR-seq (Table 1)—was previously found to be upregulated in AF, regulating structural [58,76] and electrical remodeling of atria [77]. Further, the miR-302 family was found deregulated in AF in some studies [78,79]. Additionally, among the significantly upregulated genes, SELE, as an example, has been previously associated with AF [80].

## 4. Materials and Methods

### 4.1. Collection of Human Atrial Samples and Ethical Approval Statement

The use of human tissue samples was approved by Qilu Hospital of Shandong University Research Ethical Committee (Approval#: KYLL-2021(ZM)-231). Human atrial samples were collected from patients who are undergoing surgical operations (Table 1) and stored in a tissue bank at Qilu Hospital of Shandong University.

### 4.2. Preparation of RNA Samples and mRNA Sequencing

Total RNA from frozen atrial tissue samples was extracted using Trizol Reagent (Invitrogen, Carlsbad, CA, USA) and RNA quality was determined by NanoDrop (Thermoscientific, Boston, MA, USA). Sequencing libraries were generated from 3 μg RNA using the TruSeq RNA Sample Preparation Kit (Illumina Inc., San Diego, CA, USA). For mRNA-seq, mRNA was purified from total RNA samples using magnetic beads attached to poly-T oligos. Fragmentation was performed in an Illumina proprietary fragmentation bugger using divalent cations under high temperatures. SuperScript II was used to synthesize first strand cDNA with random oligonucleotides. Subsequently, the second strand was synthesized by DNA Polymerase I and RNase H. Remaining overhangs were blunted by an exonuclease/polymerase and then enzymes were removed. Following 3’-end biotinylation, Illumina PE adapter oligos were ligated to DNA fragments to prepare for hybridization. Library fragments were purified by AMPure XP system (Beckman Coulter, Beverly, CA, USA) to select cDNA fragments with a preferred 300 bp in length. DNA fragments with adapter fragments on both ends were selectively enriched in a 15-cycle PCR reaction with Illumina PCR Primer Cocktail. Resulted products were then purified (AM Pure XP system) and quantified using a DNA quantification assay on Bioanalyzer 2100 system (Agilent). Then, RNA-seq was performed by Shanghai Personal Biotech Cp. Ltd. (Shanghai, China) on a Hiseq X platform (Illumina Inc., San Diego, CA, USA). Raw read counts were aligned to the human genome using Bowtie2 (v2.2.6). Pheatmap software package was used to perform cluster analysis on all sequenced mRNAs using R language. The Euclidean method was used for the distance measurement, and the hierarchical clustering longest distance method (Complete Linkage) was used for clustering.

### 4.3. mRNA-Seq Data Analysis

Differentially expressed genes (DEGs) were identified using DESeq (Version 1.18.0). Nominal *p*-values were adjusted by the false data discovery rate (FDR) criterion as needed. Changes in gene expression with |log_2_(FoldChange)| > 1 (or FoldChange > 1.5) and a statistical value of *p* < 0.05 were considered statistically significant. Significantly deregulated genes were then used for subsequent analyses.

Gene ontology (GO, http://geneontology.org/ (accessed on 20 September 2021)) and Kyoto Encyclopedia of Genes and Genomes (KEGG, http://www.kegg.jp/ (accessed on 20 September 2021)) pathway analyses were performed for the targets of differentially expressed miRs and genes using the DAVID database (https://david.ncifcrf.gov (accessed on 20 September 2021)). Hallmark gene-enrichment analysis was run in GSEA (www.gsea-msigdb.org (accessed on 21 March 2022)) to determine deregulated signaling pathways. Cross-tabulation analysis (Venn analysis) was performed to obtain the expression of predicted AF-related genes. The results of the raw signal analysis were then further validated by searching the literature and in vitro experiments. Cytoscape (V3.6, https://cytoscape.org/release_notes_3_6_0.html (accessed on 20 September 2021)) was used to establish the interaction network of miRNAs and target genes. Venn diagrams showing the overlay of different gene sets were generated by Venny v2.1 (https://bioinfogp.cnb.csic.es/tools/venny/ (accessed on 21 March 2022)).

The protein–protein interaction (PPI) network was constructed for genes, which are in the GSEA analysis of differentially expressed genes. Genes with a positive enrichment score of ES > 1.5 or a negative enrichment score of ES < −1.5 were selected for the PPI network. Genes with at least one interaction and a confidence score of >50 were shown in the PPI network. Positively or negatively enriched genes in the PPI network with the set criterion were searched through the molecular signatures database (MSigDB v7.4, https://www.gsea-msigdb.org/gsea/msigdb/ (accessed on 15 March 2022)) for GO and Canonical pathway analyses. The overlay of genes in the PPI network and AF-related genes in our RNA-seq data were visualized by Venny2.1. AF-related genes were obtained from the gene-disease association database (https://www.disgenet.org (accessed on 15 March 2022)) by searching the term “Atrial Fibrillation; CUI: C0004238”.

### 4.4. Preparation of RNA Samples and microRNA Sequencing

Total RNA was isolated using Trizol Reagent (Invitrogen, Carlsbad, CA, USA), and the concentration, quality, and integrity were measured using a NanoDrop spectrophotometer (Thermoscientific, Boston, MA, USA). NEBNext Multiplex Small RNA Library Prep Set for Illumina (New England Biolabs Inc., Ipswich, MA, USA) was used for constructing small RNA libraries based on the manufacturer’s instructions. Briefly, 1 μg total RNA from atrial tissue samples of sinus rhythm (SR) and AF patients was ligated to 3’ and 5’ adapters using Ligation Enzyme Mix. Ligated RNA products were reverse transcribed using Superscript II reverse transcriptase and amplified by PCR reaction. The average size of inserts was around 140–150 bp. Small RNA libraries were quality controlled and quantified using the Agilent High Sensitivity DNA Assay on a Bioanalyzer 2100 System. The small RNA library was then sequenced on NovaSeq 6000 platform (Illumina, San Diego, CA, USA) by Shanghai Personal Biotechnology Cp. Ltd. (Shanghai, China).

### 4.5. MicroRNA Sequencing Data Analysis

The quality control of raw data was performed and filtered using the Personalbio company’s self-developed script. Clean data were obtained by removing adapter and low-quality sequences. Clean reads were filtered for 18 to 36 nt in length, and deduplication was performed to obtain Unique Reads for subsequent analysis. Raw reads were aligned to the human genome by Bowtie2 (v2.2.6, https://sourceforge.net/projects/bowtie-bio/files/bowtie2/2.2.6/ (accessed on 17 June 2021)). Clean Reads were annotated with reference to the human genome using the miRDeep2 (v2.0.0.8, https://github.com/rajewsky-lab/mirdeep2 (accessed on 17 June 2021)) software. Using the miRBase database (http://www.mirbase.org/ (accessed on 17 June 2021)), unique reads were annotated with known miRs and then other non-coding RNAs. The sequences which are not annotated were analyzed using mireap (v2.0, https://sourceforge. net/projects/mireap/ (accessed on 17 June 2021)) for new miR prediction. Then, the existing annotation results were organized based on known miRNA > piRNA > rRNA > tRNA > snRNA > snoRNA > novel miRNA priority to ensure unique annotations.

Differentially expressed miRs were identified using DESeq (v1.18.0, https://www-huber.embl.de/users/anders/DESeq/ (accessed on 17 June 2021)), and transcripts with |log_2_(FoldChange)| > 1 and *p*-value < 0.05 were considered as differentially expressed. Pheatmap software package of R was used to perform bidirectional cluster analysis on all miRNAs. The distance was calculated by the Euclidean method, and genes were clustered using the hierarchical clustering longest distance method (Complete Linkage).

The target genes of differentially expressed miRs were identified using MiRanda (v3.3a, https://anaconda.org/bioconda/miranda (accessed on 17 June 2021)). The 3 ’UTR sequence of the mRNA is considered to be the target sequence of miRs. GO and KEGG enrichment analyses were performed on the target genes of differentially expressed miRs as described above for mRNA-seq (Section 4.3).

### 4.6. Cell Culture and Transfection

HEK293T cells were cultured in complete media composed of DMEM (Gibco, Grand Island, NY, USA) and 10% FBS (Gibco, Grand Island, NY, USA). Cells were passaged into 24-well plates the day before transfection and transfected at 50–70% confluency using Lipofectamine 2000 (Invitrogen, Carlsbad, CA, USA) according to the instructions of the supplier. Briefly, in separate tubes, 5 µL Lipofectamine 2000 was diluted in 150 µL OPTIMEM media (Gibco, Grand Island, NY, USA), and 5 µg miR mimics were diluted in 150 µL OPTIMEM. Following 5 min incubation at room temperature, dilutes were mixed and incubated at room temperature for 20 min to form the RNA–lipid complex. Then, the complex was added on cells with serum-free OPTIMEM media and incubated for 6 h in an incubator with 37 °C and 5% CO_2_. Once transfection was completed, serum-free cell media was replaced with complete DMEM media, and cells were harvested at 48 h post-transfection for downstream assays.

### 4.7. Gene Expression Analysis by qRT-PCR

The sequencing results were validated with the quantitative real-time PCR method. At the tissue level, total RNA was extracted from the right atrial appendage of AF and non-AF patients. The total RNA of cells and tissues were extracted, and microRNAs and mRNA were reversed transcribed to cDNA using the Reverse Transcription SparkJade MicroRNA/mRNA Reverse Transcription Kit which is the method of adding a tail (Cat#. AG0501/AG0304, SparkJade, Qingdao, China) according to the manufacturer’s instructions. qRT-PCR was performed using SYBR Green Master Mix (SparkJade, Qingdao, China) on a Bio-Rad iCycler (Hercules, CA, USA) [81]. Primer sequences for gene expression analysis are listed as followed.

H-SDC1-Forward: ACTCATCTGGCCTCAACGAC

H-SDC1-Reverse: GTGTGGGGAGTGTGAAGGTC

H-GAPDH-Forward: GCACCGTCAAGGCTGAGAAC

H-GAPDH-Reverse: TGGTGAAGACGCCAGTGGA

Hsa-miR-302d-3p Forward: ACUUUAACAUGGAGGCACUUGC

Hsa-miR-302b-3p Forward: UAAGUGCUUCCAUGUUUUAGUAG

Hsa-miR-302a-3p Forward: UAAGUGCUUCCAUGUUUUGGUGA

Hsa-U6 Forward: Purchased from Beijing Tiangen Biochemical Tech., Co., LTD (Beijing, China)

miRNA Reverse: Provided in the qPCR kit (SparkJade, Qingdao, China)

### 4.8. Protein Expression Analysis with Western Blot

For total protein collection, tissues and cells were harvested following homogenization in RIPA lysis buffer with phosphatase and protease inhibitors, followed by sonication for 10 s. Protein concentration was measured by a Biyuntian BCA protein kit (Cat# P0012, Beyotime, Shanghai, China), and protein samples were denatured in 5× loading buffer (Cat# P0015L, Beyotime, Shanghai, China) by boiling for 10 min at 95 °C. Lysates were run in 10% SDS-PAGE gels. Proteins were transferred to PVDF membranes, followed by 1 h of blocking with 10% milk solution at room temperature. Primary antibodies against the following proteins were used: human-GAPDH (1:1000, Cat#4478, Cell Signaling Tech., Danvers, MA, USA) and human-SDC1 (1:1000, Cat# EPR6456, Abcam, Cambridge, UK) overnight at 4 °C. Membranes were washed three times and incubated with HRP-conjugated anti-rabbit secondary antibodies (1:3000, Cat#7074, Cell Signaling Tech., Danvers, MA, USA) for 1–2 h at room temperature. The signal was detected using Clarity Western ECL Substrate (Bio-Rad).

### 4.9. Luciferase Assay

Luciferase assay was performed using a dual luciferase reporter assay kit (Vazyme Biotech, Nanjing, China) using the manufacturer’s guidelines. Briefly, 293T cells were split into 24-well plates the day before transfection. Cells were transfected at ~50–70% confluence with constructs carrying putative binding site of miR-302b-3p on the wild-type 3’UTR of SDC1 (AGCACTTA/GCACTTA) or the mutant 3’ UTR of SDC1 (CTACAGGC/TACAGGC) using Lipofectamine 2000 Reagent (Invitrogen, Carlsbad, CA, USA) according to the instructions by the manufacturer. DNA-lipid complexes were added to cells and media was replaced with fresh DMEM media with 10% FBS on the next day. Cells were lysed in 1X cell lysis buffer on Day3 of transfection and incubated at RT for 5 min. The lysate was spun at high speed and the supernatant transferred into new tubes for luciferase detection. Recommended amounts of Firefly or Renilla luciferase were mixed with supernatant and luminescence was detected using a luminescence detector (Jiuyu Jintai Biotech, Beijing, China).

### 4.10. Immunohistochemistry and Picrosirius Red Staining

Fresh tissue samples from sinus rhythm (SR) and atrial fibrillation (AF) patients were embedded in paraffin and immunostained as previously published [82] with modifications. Briefly, tissues were fixed in 10% formalin and embedded in paraffin. The 4 μm slices of tissues were cut from paraffin blocks and mounted on slides. After drying at RT for 30 m in, sections were baked at 45 °C overnight and deparaffinized in xylene by 2–3 times for 10 min each time. Then, they were rehydrated with a descending ethanol gradient and finally rinsed in distilled water. After antigen retrieval with 10 mM sodium citrate (pH6.0) at 92–98 °C for 10 min and inhibition of endogenous peroxidase activity with 3% H_2_O_2_ at RT for 10 min, the sections were blocked in 10% goat serum for 10 min at room temperature and subsequently incubated with SDC1 antibodies (1:500, Cat# EPR6456, Abcam, Cambridge, UK) at 4 °C overnight. The next day, the sections were incubated with secondary antibodies conjugated with HRP peroxidase at RT for 1 h, and the SDC1 signals were detected using DAB peroxidase substrate kit (Golden Bridge Biotechnology, Beijing, China). Slides were then counterstained with hematoxylin and xylene before mounting with coverslips and imaging with a microscope.

As for picrosirius staining, 4 μm of slices of paraffin-embedded tissues were cut and mounted on slides. After deparaffinizing and hydrating tissue sections as described above, they are stained with hematoxylin for 10 min and washed with water. Then, tissue sections were immersed in 0.5% Sirius red/Picric acid staining solution for 30 min at RT and rinsed with running water to remove excess stain. Sections were dehydrated with ethanol and mounted with coverslips for imaging. Images were taken with brightfield and polarized light microscopy to visualize the collagen matrix.

### 4.11. Statistical Analyses

For RNA-seq statistical analysis, we used the hypergeometric distribution method. For GO terms and KEGG pathway analyses, nominal *p* values were used or the FDR approach was applied to adjust *p*-values as needed. A two-tailed unpaired *t*-test was used for statistical analyses of qRT-PCR and Western blot results in Prism 7. For multiple groups, we used one-way ANOVA with Tukey’s test. All data were shown as the mean ± SEM, and *p* < 0.05 was considered a statistically significant change.

## 5. Conclusions

In conclusion, RNA-seq of atrial samples from AF patients identified differentially expressed small non-coding and coding transcripts. Our network analysis revealed negatively correlated pairs of miRs and mRNAs among the top deregulated transcripts. We found that the miR-302b-3p/SDC-1 axis is a potential candidate that may function in the development or sustenance of AF in patients through modulation of atrial fibrosis via TGF signaling. In the future, experimental animal models or human iPSC models may be utilized to study the mechanism of the miR-302-3p/SDC-1 axis in the pathogenesis of AF, and it may serve as a potential therapeutic target in the treatment of AF patients.

## Figures and Tables

**Table 1:** Patient Demographics.

Group	Age	Gender	Diagnosis	History of AF	Surgery	Site of Sampling
Normal	60	Female	Congenital heart disease, atrial septal defect	No AF	Atrial septal repair	Right atrium
Normal	66	Male	Tricuspid vegetations	No AF	Excision of vegetations	Right atrium
AF	64	Female	Rheumatic heart disease, mitral stenosis	Persistent AF	Mitral valve replacement	Right atrium
AF	60	Male	Rheumatic heart disease, mitral stenosis	Persistent AF	Mitral valve replacement	Right atrium
AF	57	Female	Rheumatic heart disease, mitral stenosis	Persistent AF	Mitral valve replacement	Right atrium

**Table 2:** Top deregulated microRNAs.

microRNA ID	log_2_(FoldChange)	*p*-Value
hsa-miR-302b-3p *	−1.927993099	3.52446 × 10^−5^
hsa-miR-3059-5p *	−2.439099768	0.000210321
hsa-miR-302d-3p *	−1.925669264	0.000598812
hsa-miR-378d *	−1.1161761	0.000863964
hsa-miR-516a-5p *	−2.449280109	0.001097802
hsa-miR-302a-5p *	−1.511297201	0.001691902
hsa-miR-378i *	−1.038634253	0.00180456
hsa-miR-518c-3p *	−3.007366398	0.002448495
hsa-miR-516b-5p *	−1.640951948	0.002989037
hsa-miR-5708 *	−1.848531157	0.003210251
hsa-miR-302a-3p *	−1.884247241	0.003340718
hsa-miR-378e	−1.41425312	0.004785214
hsa-miR-302c-3p	−2.078986595	0.005110454
hsa-miR-517a-3p	−2.146095895	0.006125071
hsa-miR-517b-3p	−2.146095895	0.006125071
hsa-miR-526b-5p	−4.103564723	0.00733209
hsa-miR-371a-3p	−1.330621285	0.01025266
hsa-miR-520g-3p	−1.89787358	0.01062743
hsa-miR-585-3p	−1.925975099	0.01506384
hsa-miR-372-3p	−1.232670652	0.015762249
hsa-miR-4662a-5p	−1.128104683	0.016532088
hsa-miR-378h	−1.380015786	0.018242992
hsa-miR-520c-3p	−1.84779246	0.028579751
hsa-miR-523-3p	−2.295223982	0.039597527
hsa-miR-520a-3p	−1.640624273	0.03967688
hsa-miR-519a-5p	−2.207467024	0.04264619
hsa-miR-1323	−2.430852745	0.045529514
hsa-miR-520f-3p	−1.351910815	0.046170691
hsa-miR-520b-3p	−2.29949029	0.047158202
hsa-miR-1323	−2.430852745	0.045529514
hsa-miR-146b-5p *	2.347289373	7.56934 × 10^−9^
hsa-miR-146b-3p *	2.289327335	2.21466 × 10^−7^
hsa-miR-155-5p *	1.274620071	0.00071305
hsa-miR-3690 *	1.641664997	0.004215924
hsa-miR-187-5p *	1.844437031	0.011552148
hsa-miR-187-3p *	1.028719714	0.013972754
hsa-miR-592 *	3.091036819	0.023513587
hsa-miR-212-3p *	1.148408853	0.024049925
hsa-miR-549a-3p *	2.91790048	0.026012753

* Top selected miRs for constructing a regulatory network of miR–gene pairs.

**Table 3:** Overlapping genes between predicted targets of top 11 downregulated miRs and top 50 positively enriched genes in GSEA.

Gene Name	NCBI_GI	Rank among Top 50 Upregulated Genes	miRNA ID	Rank among Downregulated miRs	miRDB Target Score (Out of 100)
FAM72A	729533	1	hsa-miR-3059-5p	2	67
KYAT1	883	3	hsa-miR-302a-5p	6	51
LRRC38	126755	4	hsa-miR-516b-5p	9	50
SDC1	6382	8	hsa-miR-302b-3p	1	96
			hsa-miR-302d-3p	3	96
			hsa-miR-302a-3p	11	96
PTCHD4	442213	9	hsa-miR-3059-5p	2	93
TYW1B	441250	10	hsa-miR-3059-5p	2	77
FCER2	2208	21	hsa-miR-516b-5p	9	82
SELE	6401	31	hsa-miR-3059-5p	2	65
FBXL16	146330	35	has-miR-3059-5p	2	63

**Table 4:** Overlapping genes between predicted targets of top 9 upregulated miRs and top 50 negatively enriched genes in GSEA.

Gene Name	NCBI_GI	Rank among Top 50 Downregulated mRNAs	miRNA ID	Rank among Upregulated miRs	miRDB Target Score (Out of 100)
FERMT1	55612	4	hsa-miR-549a-3p	9	67
SLC36A2	153201	7	hsa-miR-187-3p	6	57
GPM6B	2824	25	hsa-miR-146b-5p	1	86
			hsa-miR-155-5p	3	82
			hsa-miR-3690	4	80
CCNI2	645121	29	hsa-miR-592	7	57
MCTP2	55784	39	hsa-miR-146b-5p	1	78
GUCY1A2	2977	50	hsa-miR-187-5p	5	76

## Data Availability

All supporting data and materials are available online. RNA-seq datasets are available from the corresponding authors upon a reasonable request.

## Supplementary Materials

The following supporting information can be downloaded at: https://www.mdpi.com/article/10.3390/cells11172629/s1. Figure S1: Experimental validation of some of the selected candidate genes, which are upregulated in mRNAseq dataset, by qPCR from atrial tissue samples; Figure S2: Analysis of Top Positively Enriched Genes in GSEA; Figure S3: Analysis of Top Negatively Enriched Genes in GSEA.
