# Supplementary material for: Integrated Analysis of the microRNA–mRNA Network Predicts Potential Regulators of Atrial Fibrillation in Humans
Source: Cells. 2022 Aug 24;11(17):2629. doi: 10.3390/cells11172629 (PMC9454849; doi:10.3390/cells11172629)
Supplement: Supplementary file 1 [file cells-11-02629-s001.zip › cells-1862574-supplementary.pdf]

SUPPLEMENTAL FIGURES

Integrated Analysis of microRNA-mRNA Network Predicts Potential Regulators of Atrial Fibrillation in Human

Rong Wang, Emre Bektik*, Phraew Sakon, Xiaowei Wang, Shanying Huang, Xiangbin Meng, Mo Chen, Wenqiang Han, Jie Chen, Yanhong Wang and Jingquan Zhong*

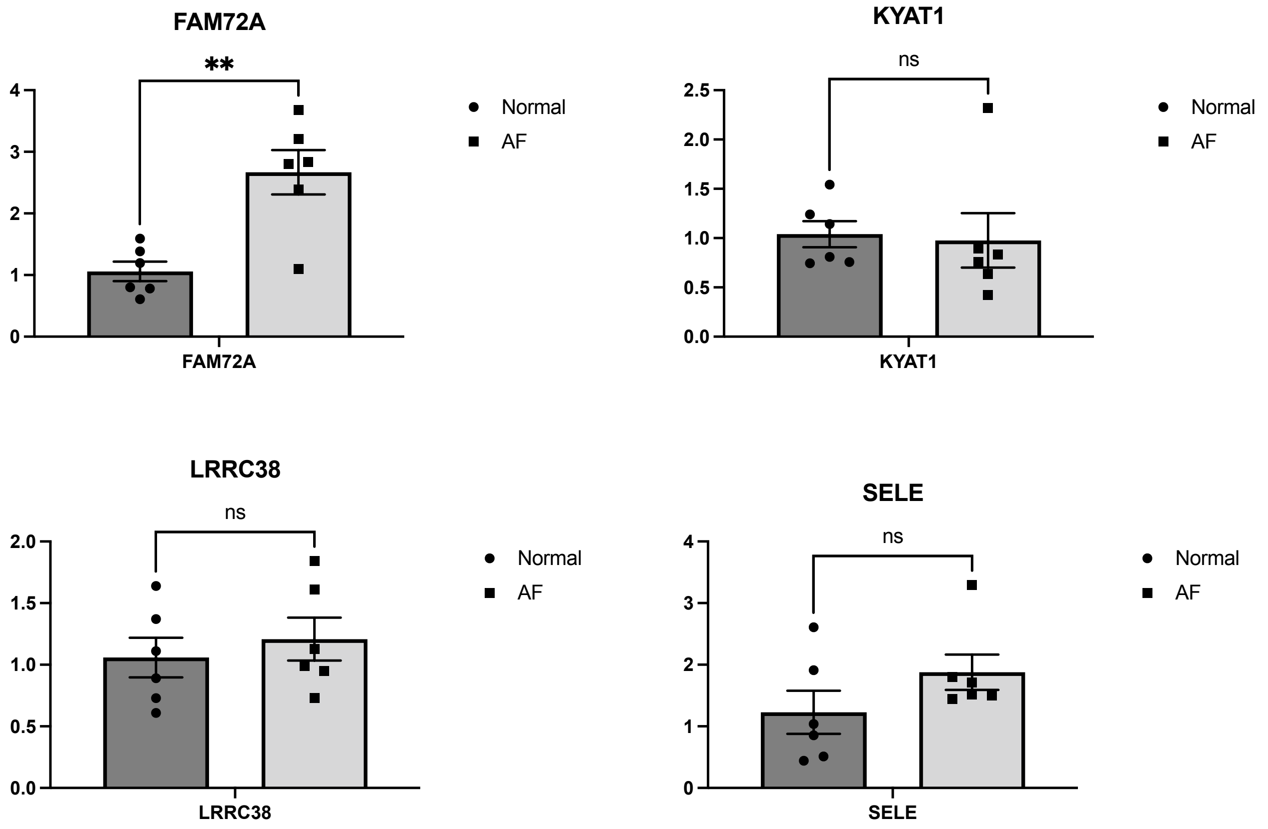

Figure S1. Experimental validation of some of the selected candidate genes, which are upregulated in mRNAseq dataset, by qPCR from atrial tissue samples.

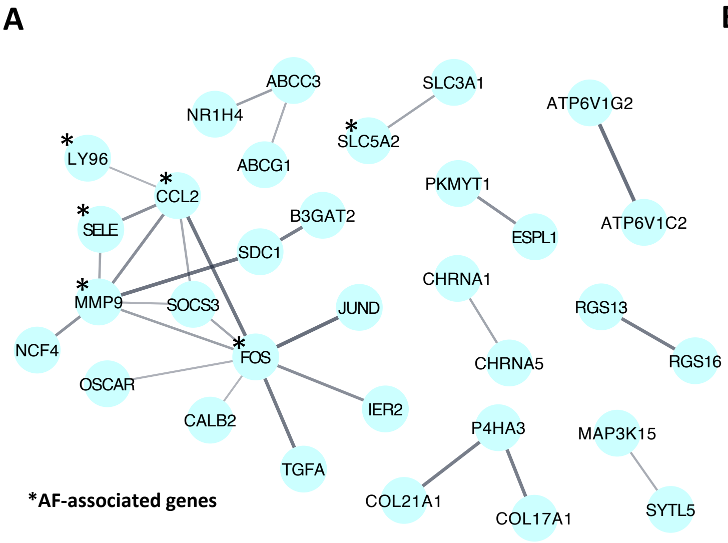

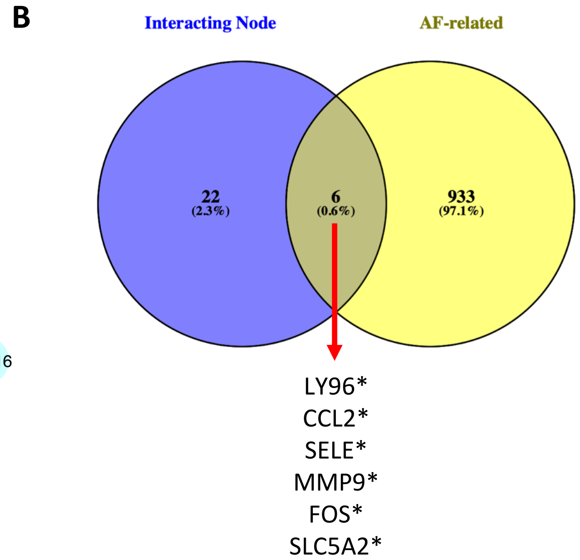

C

Gene Set Name [# Genes (K)]	Description	# Genes in Overlap (k)	k/K	p-value	FDR q-value
HALLMARK_P53_PATHWAY [200]	Genes involved in p53 pathways and networks.	4		1.1 e^{-5}	2.74 e^{-4}
HALLMARK_TNFA_SIGNALING_VIA_NFKB [200]	Genes regulated by NF-kB in response to TNF [GeneID=7124].	4		1.1 e^{-5}	2.74 e^{-4}
HALLMARK_ALLOGRAFT_REJECTION [200]	Genes up-regulated during transplant rejection.	3		3.6 e^{-4}	3 e^{-3}
HALLMARK_APICAL_JUNCTION [200]	Genes encoding components of apical junction complex.	3		3.6 e^{-4}	3 e^{-3}
HALLMARK_INFLAMMATORY_RESPONSE [200]	Genes defining inflammatory response.	3		3.6 e^{-4}	3 e^{-3}
HALLMARK_KRAS_SIGNALING_UP [200]	Genes up-regulated by KRAS activation.	3		3.6 e^{-4}	3 e^{-3}
HALLMARK_UV_RESPONSE_UP [158]	Genes up-regulated in response to ultraviolet (UV) radiation.	2		5.4 e^{-3}	3.85 e^{-2}
HALLMARK_ESTROGEN_RESPONSE_EARLY [200]	Genes defining early response to estrogen.	2		8.5 e^{-3}	4.25 e^{-2}
HALLMARK_GLYCOLYSIS [200]	Genes encoding proteins involved in glycolysis and gluconeogenesis.	2		8.5 e^{-3}	4.25 e^{-2}
HALLMARK_INTERFERON_GAMMA_RESPONSE [200]	Genes up-regulated in response to IFNG [GeneID=3458].	2		8.5 e^{-3}	4.25 e^{-2}

Entrez Gene Id	Gene Symbol	HALLMARK_P53_PATHWAY	HALLMARK_TNFA_SIGNALING_VIA_NFKB	HALLMARK_ALLOGRAFT_REJECTION	HALLMARK_APICAL_JUNCTION	HALLMARK_INFLAMMATORY_RESPONSE	HALLMARK_KRAS_SIGNALING_UP	HALLMARK_UV_RESPONSE_UP	HALLMARK_ESTROGEN_RESPONSE_EARLY	HALLMARK_GLYCOLYSIS	HALLMARK_INTERFERON_GAMMA_RESPONSE	Entrez	Ensembl	Gene Description
2353	FOS	■	■					■	■					"Fos proto-oncogene, AP-1 transcription factor subunit [Source:HGNC Symbol;Acc:HGNC:3796]"
6004	RGS16	■				■	■							regulator of G protein signaling 16 [Source:HGNC Symbol;Acc:HGNC:9997]
6382	SDC1	■								■				syndecan 1 [Source:HGNC Symbol;Acc:HGNC:10658]
7039	TGFA	■								■				transforming growth factor alpha [Source:HGNC Symbol;Acc:HGNC:11765]
6347	CCL2		■	■		■					■			C-C motif chemokine ligand 2 [Source:HGNC Symbol;Acc:HGNC:10618]
9021	SOCS3		■								■			suppressor of cytokine signaling 3 [Source:HGNC Symbol;Acc:HGNC:19391]
9592	IER2		■											immediate early response 2 [Source:HGNC Symbol;Acc:HGNC:28871]
4318	MMP9			■	■		■							matrix metallopeptidase 9 [Source:HGNC Symbol;Acc:HGNC:7176]
4689	NCF4			■										neutrophil cytosolic factor 4 [Source:HGNC Symbol;Acc:HGNC:7662]
794	CALB2				■				■					calbindin 2 [Source:HGNC Symbol;Acc:HGNC:1435]
1308	COL17A1				■									collagen type XVII alpha 1 chain [Source:HGNC Symbol;Acc:HGNC:2194]
6401	SELE					■								selectin E [Source:HGNC Symbol;Acc:HGNC:10718]
23643	LY96						■							lymphocyte antigen 96 [Source:HGNC Symbol;Acc:HGNC:17156]
1138	CHRNA5							■						cholinergic receptor nicotinic alpha 5 subunit [Source:HGNC Symbol;Acc:HGNC:1959]

Figure S2. Analysis of Top Positively Enriched Genes in GSEA. **A)** Protein-protein interaction (PPI) network of positively enriched genes with enrichment score >1.5. **B)** Overlap of hub genes in PPI network and AF-associated genes found in www.disgenet.org. **C)** Enrichment of Hallmark Pathways in PPI network in Panel A. Lower panel shows overlap matrix of genes and gene sets.

D

Gene Set Name [# Genes (K)]	Description	# Genes in Overlap (k)	k/K	p-value [?]	FDR q-value [?]
PID_ATF2_PATHWAY [59]	ATF-2 transcription factor network	4		$1.44\ e^{-7}$	$5.51\ e^{-5}$
PID_AP1_PATHWAY [70]	AP-1 transcription factor network	4		$2.89\ e^{-7}$	$5.52\ e^{-5}$
PID_FRA_PATHWAY [37]	Validated transcriptional targets of AP1 family members Fra1 and Fra2	3		$3.47\ e^{-6}$	$4.41\ e^{-4}$
PID_SYNDECAN_1_PATHWAY [46]	Syndecan-1-mediated signaling events	3		$6.74\ e^{-6}$	$6.44\ e^{-4}$
PID_AVB3_OPN_PATHWAY [31]	Osteopontin-mediated events	2		$2.8\ e^{-4}$	$2.14\ e^{-2}$
PID_GMCSF_PATHWAY [36]	GMCSF-mediated signaling events	2		$3.78\ e^{-4}$	$2.16\ e^{-2}$
PID_IL23_PATHWAY [37]	IL23-mediated signaling events	2		$4\ e^{-4}$	$2.16\ e^{-2}$
PID_FOXM1_PATHWAY [40]	FOXM1 transcription factor network	2		$4.67\ e^{-4}$	$2.16\ e^{-2}$
KEGG_ABC_TRANSPORTERS [44]	ABC transporters	2		$5.66\ e^{-4}$	$2.16\ e^{-2}$
PID_ERBB2_ERBB3_PATHWAY [44]	ErbB2/ErbB3 signaling events	2		$5.66\ e^{-4}$	$2.16\ e^{-2}$
PID_IL6_7_PATHWAY [47]	IL6-mediated signaling events	2		$6.45\ e^{-4}$	$2.24\ e^{-2}$
KEGG_VIBRIO_CHOLERAE_INFECTION [54]	Vibrio cholerae infection	2		$8.51\ e^{-4}$	$2.41\ e^{-2}$
PID_FGF_PATHWAY [55]	FGF signaling pathway	2		$8.83\ e^{-4}$	$2.41\ e^{-2}$
PID_IL2_1PATHWAY [55]	IL2-mediated signaling events	2		$8.83\ e^{-4}$	$2.41\ e^{-2}$
PID_LYSOPHOSPHOLIPID_PATHWAY [65]	LPA receptor mediated events	2		$1.23\ e^{-3}$	$3.13\ e^{-2}$
KEGG_EPITHELIAL_CELL_SIGNALING_IN_HELICOBACTER_PYLORI_INFECTION [68]	Epithelial cell signaling in Helicobacter pylori infection	2		$1.35\ e^{-3}$	$3.21\ e^{-2}$
KEGG_LEISHMANIA_INFECTION [72]	Leishmania infection	2		$1.51\ e^{-3}$	$3.38\ e^{-2}$
PID_AVB3_INTEGRIN_PATHWAY [74]	Integrins in angiogenesis	2		$1.59\ e^{-3}$	$3.38\ e^{-2}$
PID_REG_GR_PATHWAY [82]	Glucocorticoid receptor regulatory network	2		$1.95\ e^{-3}$	$3.92\ e^{-2}$
KEGG_PATHWAYS_IN_CANCER [325]	Pathways in cancer	3		$2.17\ e^{-3}$	$4.14\ e^{-2}$

Entrez Gene Id	Gene Symbol	PID_ATF2_PATHWAY	PID_AP1_PATHWAY	PID_FRA_PATHWAY	PID_SYNDECAN_1_PATHWAY	PID_AVB3_OPN_PATHWAY	PID_GMCSF_PATHWAY	PID_IL23_PATHWAY	PID_FOXM1_PATHWAY	KEGG_ABC_TRANSPORTERS	PID_ERBB2_ERBB3_PATHWAY	PID_IL6_7_PATHWAY	KEGG_VIBRIO_CHOLERAE_INFECTION	PID_FGF_PATHWAY	PID_IL2_1PATHWAY	PID_LYSOPHOSPHOLIPID_PATHWAY	KEGG_EPITHELIAL_CELL_SIGNALING_IN_HELICOBACTER_PYLORI_INFECTION	KEGG_LEISHMANIA_INFECTION	PID_AVB3_INTEGRIN_PATHWAY	PID_REG_GR_PATHWAY	KEGG_PATHWAYS_IN_CANCER	Entrez	Ensembl	Gene Description
3727	JUND	■	■	■																				"JunD proto-oncogene, AP-1 transcription factor subunit [Source:HGNC Symbol;Acc:HGNC:6206]"
2353	FOS	■	■			■	■		■		■	■		■	■	■		■		■	■			"Fos proto-oncogene, AP-1 transcription factor subunit [Source:HGNC Symbol;Acc:HGNC:3796]"
9021	SOCS3	■						■				■			■									suppressor of cytokine signaling 3 [Source:HGNC Symbol;Acc:HGNC:19391]
6401	SELE	■																		■				selectin E [Source:HGNC Symbol;Acc:HGNC:10718]
4318	MMP9		■	■	■	■								■		■					■			matrix metallopeptidase 9 [Source:HGNC Symbol;Acc:HGNC:7176]
6347	CCL2		■	■			■	■																C-C motif chemokine ligand 2 [Source:HGNC Symbol;Acc:HGNC:10618]
6382	SDC1				■														■					syndecan 1 [Source:HGNC Symbol;Acc:HGNC:10658]
1308	COL17A1				■														■					collagen type XVII alpha 1 chain [Source:HGNC Symbol;Acc:HGNC:2194]
7039	TGFA								■												■			transforming growth factor alpha [Source:HGNC Symbol;Acc:HGNC:11765]
8714	ABCC3									■														ATP binding cassette subfamily C member 3 [Source:HGNC Symbol;Acc:HGNC:54]
9619	ABCG1									■														ATP binding cassette subfamily G member 1 [Source:HGNC Symbol;Acc:HGNC:73]
1134	CHRNA1										■													cholinergic receptor nicotinic alpha 1 subunit [Source:HGNC Symbol;Acc:HGNC:1955]
245973	ATP6V1C2												■				■							ATPase H+ transporting V1 subunit C2 [Source:HGNC Symbol;Acc:HGNC:18264]
534	ATP6V1G2												■				■							ATPase H+ transporting V1 subunit G2 [Source:HGNC Symbol;Acc:HGNC:862]
4689	NCF4																	■						neutrophil cytosolic factor 4 [Source:HGNC Symbol;Acc:HGNC:7662]

Figure S2 Cont. Analysis of Top Positively Enriched Genes in GSEA. **D)** Pathway enrichment of hub genes in PPI network in Panel A. Lower panel shows overlap matrix of genes and gene sets.

E

GO terms– Biological Process

Gene Set Name [# Genes (K)]	Description	# Genes in Overlap (k)	k/K	p-value	FDR q-value
GOBP_RESPONSE_TO_OXYGEN_CONTAINING_COM COMPOUND [1654]	Any process that results in a change in state or activity of a cell or an organism (in terms of movement, secretion, enzyme production, gene expression, etc.) as a result of an oxygen-containing compound stimulus. [GOC:pr, GOC:TermGenie]	11		$3.12\ e^{-8}$	$2.34\ e^{-4}$
GOBP_INFLAMMATORY_RESPONSE [773]	The immediate defensive reaction (by vertebrate tissue) to infection or injury caused by chemical or physical agents. The process is characterized by local vasodilation, extravasation of plasma into intercellular spaces and accumulation of white blood cells and macrophages. [GO_REF:0000022, ISBN:0198506732]	8		$1.23\ e^{-7}$	$4.34\ e^{-4}$
GOBP_TRANSMEMBRANE_TRANSPORT [1605]	The process in which a solute is transported across a lipid bilayer, from one side of a membrane to the other. [GOC:dph, GOC:jid]	10		$2.81\ e^{-7}$	$4.34\ e^{-4}$
GOBP_RESPONSE_TO_MOLECULE_OF_BACTERIAL IAL_ORIGIN [346]	Any process that results in a change in state or activity of an organism (in terms of movement, secretion, enzyme production, gene expression, etc.) as a result of a stimulus by molecules of bacterial origin such as peptides derived from bacterial flagellin. [GOC:rl, GOC:sm]	6		$2.87\ e^{-7}$	$4.34\ e^{-4}$
GOBP_RESPONSE_TO_LIPID [865]	Any process that results in a change in state or activity of a cell or an organism (in terms of movement, secretion, enzyme production, gene expression, etc.) as a result of a lipid stimulus. [GOC:sl]	8		$2.9\ e^{-7}$	$4.34\ e^{-4}$
GOBP_ENZYME_LINKED_RECEPTOR_PROTEIN_SI _SIGNALING_PATHWAY [1072]	Any series of molecular signals initiated by the binding of an extracellular ligand to a receptor on the surface of the target cell, where the receptor possesses catalytic activity or is closely associated with an enzyme such as a protein kinase, and ending with regulation of a downstream cellular process, e.g. transcription. [GOC:mah, GOC:signaling, ISBN:0815316194]	8		$1.45\ e^{-6}$	$1.6\ e^{-3}$
GOBP_TRANSMEMBRANE_RECEPTOR_PROTEIN_TY _TYROSINE_KINASE_SIGNALING_PATHWAY [737]	A series of molecular signals initiated by the binding of an extracellular ligand to a receptor on the surface of the target cell where the receptor possesses tyrosine kinase activity, and ending with regulation of a downstream cellular process, e.g. transcription. [GOC:ceb, GOC:signaling]	7		$1.5\ e^{-6}$	$1.6\ e^{-3}$
GOBP_RESPONSE_TO_NITROGEN_COMPOUND [1117]	Any process that results in a change in state or activity of a cell or an organism (in terms of movement, secretion, enzyme production, gene expression, etc.) as a result of a nitrogen compound stimulus. [GOC:pr, GOC:TermGenie]	8		$1.97\ e^{-6}$	$1.84\ e^{-3}$
GOBP_CELLULAR_RESPONSE_TO_OXYGEN_CONTA NTAINING_COMPOUND [1182]	Any process that results in a change in state or activity of a cell (in terms of movement, secretion, enzyme production, gene expression, etc.) as a result of an oxygen-containing compound stimulus. [GOC:pr, GOC:TermGenie]	8		$3\ e^{-6}$	$2.25\ e^{-3}$
GOBP_RESPONSE_TO_PEPTIDE [521]	Any process that results in a change in state or activity of a cell or an organism (in terms of movement, secretion, enzyme production, gene expression, etc.) as a result of a peptide stimulus. [GOC:pr, GOC:TermGenie]	6		$3.08\ e^{-6}$	$2.25\ e^{-3}$
GOBP_RESPONSE_TO_ENDOGENOUS_STIMULUS [1624]	Any process that results in a change in state or activity of a cell or an organism (in terms of movement, secretion, enzyme production, gene expression, etc.) as a result of a stimulus arising within the organism. [GOC:sm]	9		$3.31\ e^{-6}$	$2.25\ e^{-3}$
GOBP_RESPONSE_TO_HORMONE [876]	Any process that results in a change in state or activity of a cell or an organism (in terms of movement, secretion, enzyme production, gene expression, etc.) as a result of a hormone stimulus. [GOC:jl]	7		$4.68\ e^{-6}$	$2.91\ e^{-3}$
GOBP_INSULIN_RECEPTOR_SIGNALING_PATHWA HWAY [143]	The series of molecular signals generated as a consequence of the insulin receptor binding to insulin. [GOC:ceb]	4		$5.05\ e^{-6}$	$2.91\ e^{-3}$
GOBP_GLYCOSIDE_TRANSPORT [5]	The directed movement of a glycoside into, out of or within a cell, or between cells, by means of some agent such as a transporter or pore. [GOC:pr, GOC:TermGenie]	2		$6.1\ e^{-6}$	$3.26\ e^{-3}$
GOBP_CELLULAR_HOMEOSTASIS [937]	Any process involved in the maintenance of an internal steady state at the level of the cell. [GOC:isa_complete, GOC:jl, ISBN:0395825172]	7		$7.26\ e^{-6}$	$3.62\ e^{-3}$
GOBP_SECRETION [1464]	The controlled release of a substance by a cell or a tissue. [GOC:ai]	8		$1.43\ e^{-5}$	$6.7\ e^{-3}$

GOBP_RESPONSE_TO_BACTERIUM [717]	Any process that results in a change in state or activity of a cell or an organism (in terms of movement, secretion, enzyme production, gene expression, etc.) as a result of a stimulus from a bacterium. [GOC:hb]	6		$1.89\ e^{-5}$	$8.34\ e^{-3}$
GOBP_RESPONSE_TO_PEPTIDE_HORMONE [435]	Any process that results in a change in state or activity of a cell or an organism (in terms of movement, secretion, enzyme production, gene expression, etc.) as a result of a peptide hormone stimulus. A peptide hormone is any of a class of peptides that are secreted into the blood stream and have endocrine functions in living animals. [PMID:11027914, PMID:15134857, Wikipedia:Peptide_hormone]	5		$2.26\ e^{-5}$	$9.41\ e^{-3}$
GOBP_CELLULAR_RESPONSE_TO_INSULIN_STIM TIMULUS [226]	Any process that results in a change in state or activity of a cell (in terms of movement, secretion, enzyme production, gene expression, etc.) as a result of an insulin stimulus. Insulin is a polypeptide hormone produced by the islets of Langerhans of the pancreas in mammals, and by the homologous organs of other organisms. [GOC:mah, ISBN:0198506732]	4		$3.06\ e^{-5}$	$1.2\ e^{-2}$
GOBP_RESPONSE_TO_CYTOKINE [1188]	Any process that results in a change in state or activity of a cell or an organism (in terms of movement, secretion, enzyme production, gene expression, etc.) as a result of a cytokine stimulus. [GOC:sl]	7		$3.35\ e^{-5}$	$1.25\ e^{-2}$
GOBP_CYTOKINE_MEDIATED_SIGNALING_PATHW THWAY [803]	A series of molecular signals initiated by the binding of a cytokine to a receptor on the surface of a cell, and ending with regulation of a downstream cellular process, e.g. transcription. [GOC:mah, GOC:signaling, PMID:19295629]	6		$3.57\ e^{-5}$	$1.25\ e^{-2}$
GOBP_NEGATIVE_REGULATION_OF_RESPONSE_T E_TO_STIMULUS [1673]	Any process that stops, prevents, or reduces the frequency, rate or extent of a response to a stimulus. Response to stimulus is a change in state or activity of a cell or an organism (in terms of movement, secretion, enzyme production, gene expression, etc.) as a result of a stimulus. [GOC:jid]	8		$3.73\ e^{-5}$	$1.25\ e^{-2}$
GOBP_CARBOHYDRATE_DERIVATIVE_TRANSPORT [82]	The directed movement of a carbohydrate derivative into, out of or within a cell, or between cells, by means of some agent such as a transporter or pore. [GOC:bf, GOC:jl, GOC:TermGenie]	3		$3.86\ e^{-5}$	$1.25\ e^{-2}$
GOBP_DEFENSE_RESPONSE [1790]	Reactions, triggered in response to the presence of a foreign body or the occurrence of an injury, which result in restriction of damage to the organism attacked or prevention/recovery from the infection caused by the attack. [GOC:go_curators]	8		$6.02\ e^{-5}$	$1.88\ e^{-2}$
GOBP_RESPONSE_TO_INSULIN [278]	Any process that results in a change in state or activity of a cell or an organism (in terms of movement, secretion, enzyme production, gene expression, etc.) as a result of an insulin stimulus. Insulin is a polypeptide hormone produced by the islets of Langerhans of the pancreas in mammals, and by the homologous organs of other organisms. [GOC:mah, ISBN:0198506732]	4		$6.84\ e^{-5}$	$2.05\ e^{-2}$
GOBP_REGULATION_OF_MITOTIC_NUCLEAR_DIV DIVISION [109]	Any process that modulates the frequency, rate or extent of mitosis. [GOC:go_curators]	3		$9.01\ e^{-5}$	$2.59\ e^{-2}$
GOBP_HOMEOSTATIC_PROCESS [1912]	Any biological process involved in the maintenance of an internal steady state. [GOC:jl, ISBN:0395825172]	8		$9.55\ e^{-5}$	$2.62\ e^{-2}$
GOBP_NEGATIVE_REGULATION_OF_SIGNALING [1409]	Any process that stops, prevents, or reduces the frequency, rate or extent of a signaling process. [GOC:mtg_signal]	7		$9.81\ e^{-5}$	$2.62\ e^{-2}$
GOBP_CELLULAR_RESPONSE_TO_HORMONE_STIM TIMULUS [619]	Any process that results in a change in state or activity of a cell (in terms of movement, secretion, enzyme production, gene expression, etc.) as a result of a hormone stimulus. [GOC:mah]	5		$1.2\ e^{-4}$	$3.1\ e^{-2}$
GOBP_CELLULAR_RESPONSE_TO_PEPTIDE_HORM ORMONE_STIMULUS [325]	Any process that results in a change in state or activity of a cell (in terms of movement, secretion, enzyme production, gene expression, etc.) as a result of a peptide hormone stimulus. A peptide hormone is any of a class of peptides that are secreted into the blood stream and have endocrine functions in living animals. [GOC:mah]	4		$1.25\ e^{-4}$	$3.11\ e^{-2}$
GOBP_REGULATION_OF_RESPONSE_TO_EXTERNA RNAL_STIMULUS [1036]	Any process that modulates the frequency, rate or extent of a response to an external stimulus. [GOC:mah]	6		$1.45\ e^{-4}$	$3.5\ e^{-2}$
GOBP_RESPONSE_TO_METAL_ION [351]	Any process that results in a change in state or activity of a cell or an organism (in terms of movement, secretion, enzyme production, gene expression, etc.) as a result of a metal ion stimulus. [GOC:sm]	4		$1.68\ e^{-4}$	$3.85\ e^{-2}$
GOBP_REGULATION_OF_NUCLEAR_DIVISION [135]	Any process that modulates the frequency, rate or extent of nuclear division, the partitioning of the nucleus and its genetic information. [GOC:ai]	3		$1.7\ e^{-4}$	$3.85\ e^{-2}$
GOBP_REGULATION_OF_INFLAMMATORY_RESPON PONSE [366]	Any process that modulates the frequency, rate or extent of the inflammatory response, the immediate defensive reaction (by vertebrate tissue) to infection or injury caused by chemical or physical agents. [GOC:ai]	4		$1.97\ e^{-4}$	$4.33\ e^{-2}$
GOBP_RESPONSE_TO_CALCIUM_ION [144]	Any process that results in a change in state or activity of a cell or an organism (in terms of movement, secretion, enzyme production, gene expression, etc.) as a result of a calcium ion stimulus. [GOC:ai]	3		$2.05\ e^{-4}$	$4.39\ e^{-2}$

GOBP_RESPONSE_TO_BIOTIC_STIMULUS [1606]	Any process that results in a change in state or activity of a cell or an organism (in terms of movement, secretion, enzyme production, gene expression, etc.) as a result of a biotic stimulus, a stimulus caused or produced by a living organism. [GOC:hb]	7		$2.2\ e^{-4}$	$4.48\ e^{-2}$
GOBP_PHAGOSOME_ACIDIFICATION [28]	Any process that reduces the pH of the phagosome, measured by the concentration of the hydrogen ion. [GOC:kmv, GOC:tb]	2		$2.28\ e^{-4}$	$4.48\ e^{-2}$
GOBP_SYNAPTIC_TRANSMISSION_CHOLINERGIC [28]	The vesicular release of acetylcholine from a presynapse, across a chemical synapse, the subsequent activation of dopamine receptors at the postsynapse of a target cell (neuron, muscle, or secretory cell) and the effects of this activation on the postsynaptic membrane potential and ionic composition of the postsynaptic cytosol. This process encompasses both spontaneous and evoked release of neurotransmitter and all parts of synaptic vesicle exocytosis. Evoked transmission starts with the arrival of an action potential at the presynapse. [GOC:dos, Wikipedia:Cholinergic]	2		$2.28\ e^{-4}$	$4.48\ e^{-2}$
GOBP_CELLULAR_RESPONSE_TO_PEPTIDE [391]	Any process that results in a change in state or activity of a cell (in terms of movement, secretion, enzyme production, gene expression, etc.) as a result of a peptide stimulus. [GOC:pr, GOC:TermGenie]	4		$2.53\ e^{-4}$	$4.85\ e^{-2}$
GOBP_CATION_TRANSPORT [1155]	The directed movement of cations, atoms or small molecules with a net positive charge, into, out of or within a cell, or between cells, by means of some agent such as a transporter or pore. [GOC:ai]	6		$2.61\ e^{-4}$	$4.89\ e^{-2}$

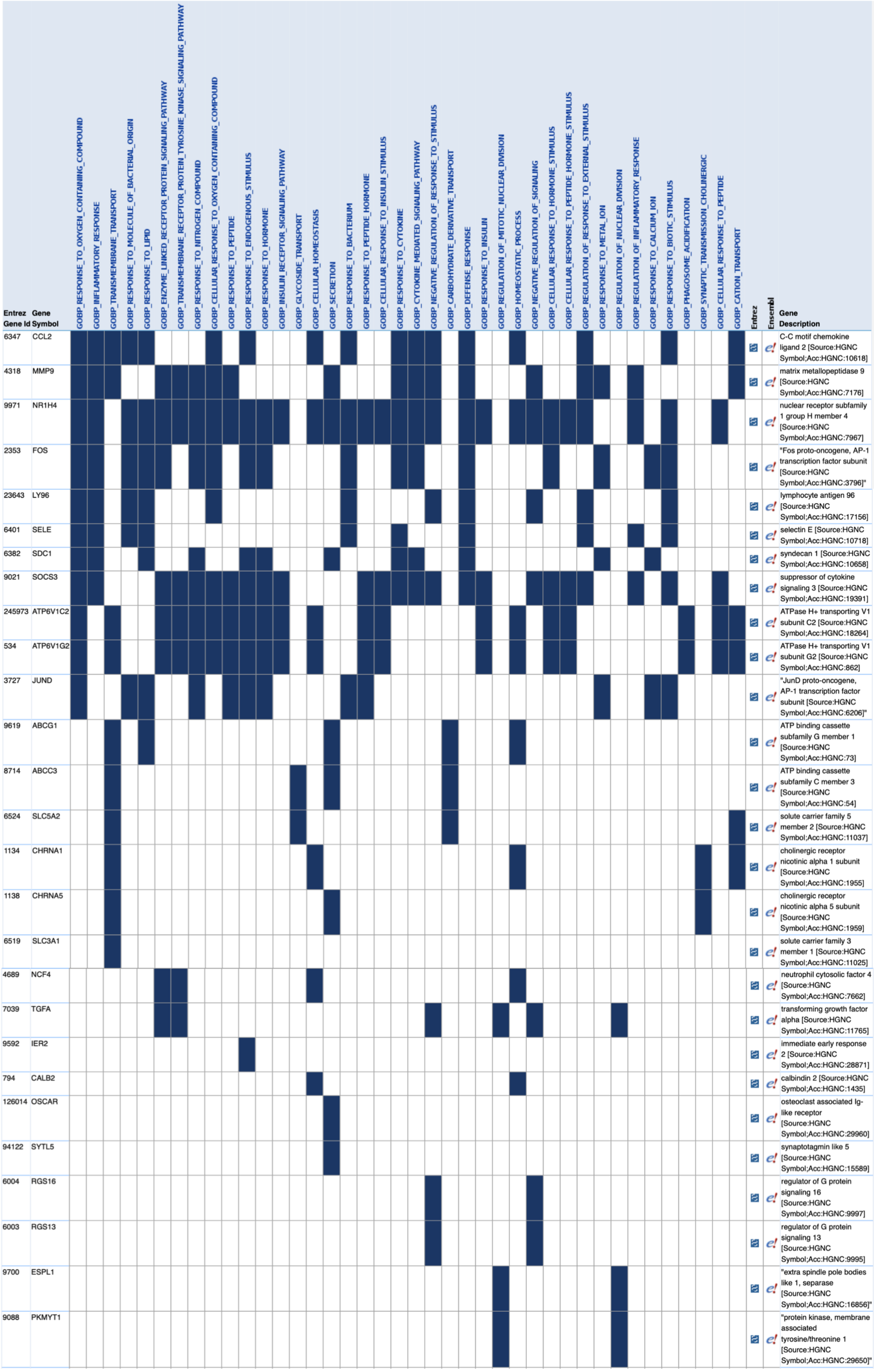

Figure S2 Cont. E) GO-term (Biological Process) analysis of hub genes in PPI network and gene/gene-set overlap matrix.

F

GO terms- Cellular Compartment

Gene Set Name [# Genes (K)]	Description	# Genes in Overlap (k)	k/K	p-value	FDR q-value
GOCC_INTRINSIC_COMPONENT_OF_PLASMA_MEMBRANE [1730]	The component of the plasma membrane consisting of the gene products and protein complexes having either part of their peptide sequence embedded in the hydrophobic region of the membrane or some other covalently attached group such as a GPI anchor that is similarly embedded in the membrane. [GOC:dos, GOC:mah]	8		4.73 e^{-5}	2.31 e^{-2}
GOCC_MEMBRANE_PROTEIN_COMPLEX [1319]	Any protein complex that is part of a membrane. [GOC:dos]	7		6.49 e^{-5}	2.31 e^{-2}
GOCC_ACETYLCHOLINE_GATED_CHANNEL_COMPLEX [16]	A homo- or hetero-pentameric protein complex that forms a transmembrane channel through which ions may pass in response to acetylcholine binding. [GOC:bf, GOC:mah, PMID:12381728, PMID:15579462]	2		7.27 e^{-5}	2.31 e^{-2}
GOCC_VACUOLAR_PROTON_TRANSPORTING_V_TYPE_ATPASE_COMPLEX [18]	A proton-transporting two-sector ATPase complex found in the vacuolar membrane, where it acts as a proton pump to mediate acidification of the vacuolar lumen. [GOC:mah, ISBN:0716743663, PMID:16449553]	2		9.27 e^{-5}	2.31 e^{-2}
GOCC_PROTON_TRANSPORTING_V_TYPE_ATPASE_COMPLEX [27]	A proton-transporting two-sector ATPase complex that couples ATP hydrolysis to the transport of protons across a concentration gradient. The resulting transmembrane electrochemical potential of H+ is used to drive a variety of (i) secondary active transport systems via H+-dependent symporters and antiporters and (ii) channel-mediated transport systems. The complex comprises a membrane sector (V0) that carries out proton transport and a cytoplasmic compartment sector (V1) that catalyzes ATP hydrolysis. V-type ATPases are found in the membranes of organelles such as vacuoles, endosomes, and lysosomes, and in the plasma membrane. [GOC:mah, ISBN:0716743663, PMID:16449553]	2		2.12 e^{-4}	4.22 e^{-2}

Entrez Gene Id	Gene Symbol	GOCC_INTRINSIC_COMPONENT_OF_PLASMA_MEMBRANE	GOCC_MEMBRANE_PROTEIN_COMPLEX	GOCC_ACETYLCHOLINE_GATED_CHANNEL_COMPLEX	GOCC_VACUOLAR_PROTON_TRANSPORTING_V_TYPE_ATPASE_COMPLEX	GOCC_PROTON_TRANSPORTING_V_TYPE_ATPASE_COMPLEX	Entrez	Ensembl	Gene Description
1134	CHRNA1	■	■	■					cholinergic receptor nicotinic alpha 1 subunit [Source:HGNC Symbol;Acc:HGNC:1955]
1138	CHRNA5	■	■	■					cholinergic receptor nicotinic alpha 5 subunit [Source:HGNC Symbol;Acc:HGNC:1959]
4689	NCF4	■	■						neutrophil cytosolic factor 4 [Source:HGNC Symbol;Acc:HGNC:7662]
23643	LY96	■	■						lymphocyte antigen 96 [Source:HGNC Symbol;Acc:HGNC:17156]
6519	SLC3A1	■							solute carrier family 3 member 1 [Source:HGNC Symbol;Acc:HGNC:11025]
6401	SELE	■							selectin E [Source:HGNC Symbol;Acc:HGNC:10718]
6382	SDC1	■							syndecan 1 [Source:HGNC Symbol;Acc:HGNC:10658]
1308	COL17A1	■							collagen type XVII alpha 1 chain [Source:HGNC Symbol;Acc:HGNC:2194]
534	ATP6V1G2		■		■	■			ATPase H+ transporting V1 subunit G2 [Source:HGNC Symbol;Acc:HGNC:862]
245973	ATP6V1C2		■		■	■			ATPase H+ transporting V1 subunit C2 [Source:HGNC Symbol;Acc:HGNC:18264]
9021	SOCS3		■						suppressor of cytokine signaling 3 [Source:HGNC Symbol;Acc:HGNC:19391]

Figure S2 Cont. F) GO-term (Cellular Compartment) analysis of hub genes in PPI network and gene/gene-set overlap matrix.

G GO terms– Molecular Function

Gene Set Name [# Genes (K)]	Description	# Genes in Overlap (k)	k/K	p-value	FDR q-value
GOMF_TRANSPORTER_ACTIVITY [1168]	Enables the directed movement of substances (such as macromolecules, small molecules, ions) into, out of or within a cell, or between cells. [GOC:ai, GOC:dgf]	8		$2.75\ e^{-6}$	$4.69\ e^{-3}$
GOMF_ION_TRANSMEMBRANE_TRANSPORTER_ACT ACTIVITY [941]	Enables the transfer of an ion from one side of a membrane to the other. [GOC:dgf, GOC:mtg_transport, ISBN:0815340729]	7		$7.47\ e^{-6}$	$5.2\ e^{-3}$
GOMF_ATPASE_COUPLED_LIPID_TRANSMEMBRAN RANE_TRANSPORTER_ACTIVITY [6]	Enables the transfer of a solute or solutes from one side of a membrane to the other according to the reaction: ATP + H2O + lipid(in) = ADP + phosphate + lipid(out). [GOC:BHF, GOC:rl]	2		$9.14\ e^{-6}$	$5.2\ e^{-3}$
GOMF_PRIMARY_ACTIVE_TRANSMEMBRANE_TRAN RANSPORTER_ACTIVITY [62]	Enables the transfer of a solute from one side of a membrane to the other, up the solute's concentration gradient, by binding the solute and undergoing a series of conformational changes. Transport works equally well in either direction and is powered by a primary energy source, directly using ATP. Primary energy sources known to be coupled to transport are chemical, electrical and solar sources. [GOC:mtg_transport, ISBN:0815340729, TC:3.-.-.-.-]	3		$1.66\ e^{-5}$	$7.11\ e^{-3}$
GOMF_ACETYLCHOLINE_GATED_CATION_SELECTIVE_CHANNEL_ACTIVITY [16]	Selectively enables the transmembrane transfer of a cation by a channel that opens upon binding acetylcholine. [GOC:mah, PMID:2466967]	2		$7.27\ e^{-5}$	$2.49\ e^{-2}$
GOMF_ACTIVE_TRANSMEMBRANE_TRANSPORTER_ ER_ACTIVITY [302]	Enables the transfer of a specific substance or related group of substances from one side of a membrane to the other, up the solute's concentration gradient. The transporter binds the solute and undergoes a series of conformational changes. Transport works equally well in either direction. [GOC:mtg_transport, ISBN:0815340729]	4		$9.41\ e^{-5}$	$2.68\ e^{-2}$
GOMF_ABC_TYPE_TRANSPORTER_ACTIVITY [22]	Primary active transporter characterized by two nucleotide-binding domains and two transmembrane domains. Uses the energy generated from ATP hydrolysis to drive the transport of a substance across a membrane. [PMID:26517899]	2		$1.4\ e^{-4}$	$2.98\ e^{-2}$
GOMF_ACETYLCHOLINE_RECEPTOR_ACTIVITY [22]	Combining with acetylcholine and transmitting the signal from one side of the membrane to the other to initiate a change in cell activity. [GOC:jl, GOC:signaling]	2		$1.4\ e^{-4}$	$2.98\ e^{-2}$
GOMF_EXCITATORY_EXTRACELLULAR_LIGAND_G D_GATED_ION_CHANNEL_ACTIVITY [29]	Enables the transmembrane transfer of an ion by a channel that opens when a specific extracellular ligand has been bound by the channel complex or one of its constituent parts, where channel opening contributes to an increase in membrane potential. [GOC:mah, ISBN:0323037070]	2		$2.45\ e^{-4}$	$4.64\ e^{-2}$

Entrez Gene Id	Gene Symbol	GOMF_TRANSPORTER_ACTIVITY	GOMF_ION_TRANSMEMBRANE_TRANSPORTER_ACTIVITY	GOMF_ATPASE_COUPLED_LIPID_TRANSMEMBRANE_TRANSPORTER_ACTIVITY	GOMF_PRIMARY_ACTIVE_TRANSMEMBRANE_TRANSPORTER_ACTIVITY	GOMF_ACETYLCHOLINE_GATED_CATION_SELECTIVE_CHANNEL_ACTIVITY	GOMF_ACTIVE_TRANSMEMBRANE_TRANSPORTER_ACTIVITY	GOMF_ABC_TYPE_TRANSPORTER_ACTIVITY	GOMF_ACETYLCHOLINE_RECEPTOR_ACTIVITY	GOMF_EXCITATORY_EXTRACELLULAR_LIGAND_GATED_ION_CHANNEL_ACTIVITY	Entrez	Ensembl	Gene Description
9619	ABCG1	■	■	■	■		■	■					ATP binding cassette subfamily G member 1 [Source:HGNC Symbol;Acc:HGNC:73]
8714	ABCC3	■	■	■	■		■	■					ATP binding cassette subfamily C member 3 [Source:HGNC Symbol;Acc:HGNC:54]
1134	CHRNA1	■	■			■			■	■			cholinergic receptor nicotinic alpha 1 subunit [Source:HGNC Symbol;Acc:HGNC:1955]
1138	CHRNA5	■	■			■			■	■			cholinergic receptor nicotinic alpha 5 subunit [Source:HGNC Symbol;Acc:HGNC:1959]
6524	SLC5A2	■	■				■						solute carrier family 5 member 2 [Source:HGNC Symbol;Acc:HGNC:11037]
6519	SLC3A1	■	■										solute carrier family 3 member 1 [Source:HGNC Symbol;Acc:HGNC:11025]
245973	ATP6V1C2	■	■										ATPase H+ transporting V1 subunit C2 [Source:HGNC Symbol;Acc:HGNC:18264]
534	ATP6V1G2	■			■		■						ATPase H+ transporting V1 subunit G2 [Source:HGNC Symbol;Acc:HGNC:862]

Figure S2 Cont. G) GO-term (Molecular Function) analysis of hub genes in PPI network and gene/gene-set overlap matrix.

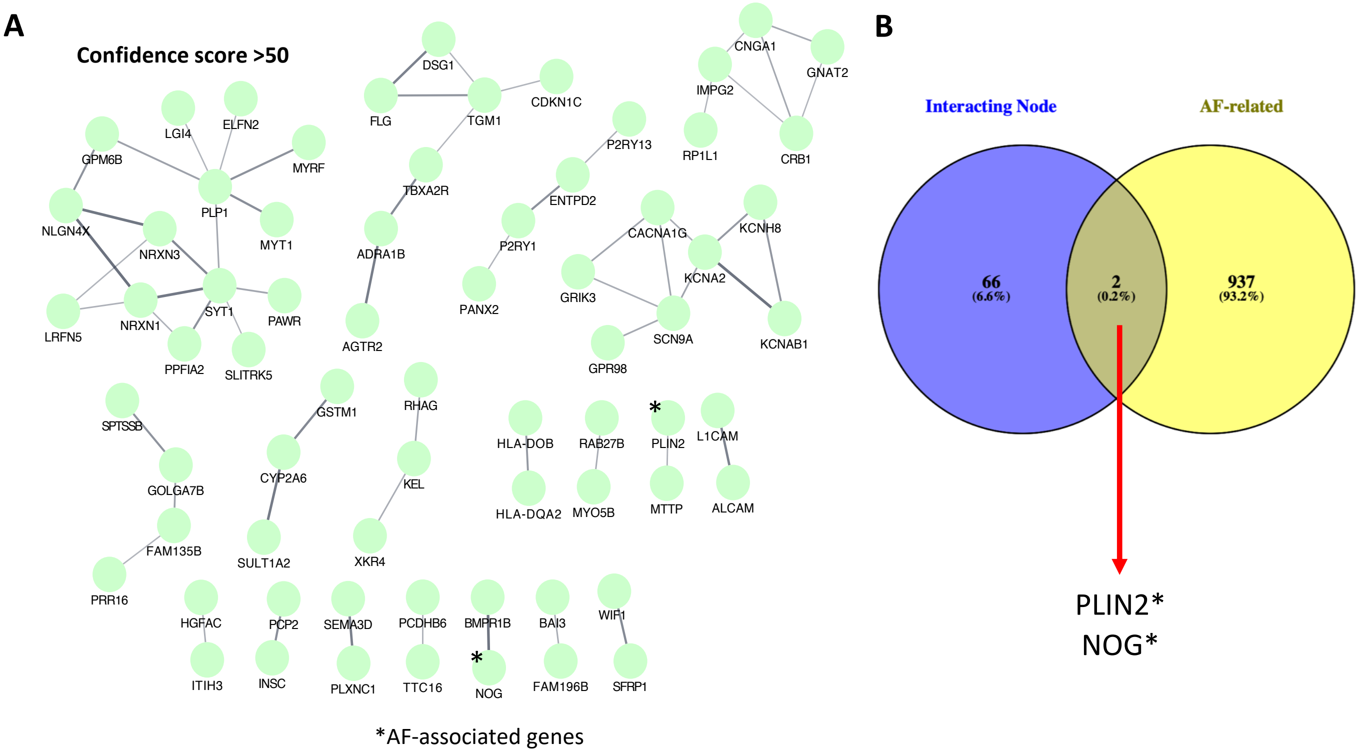

C

Gene Set Name [# Genes (K)]	Description	# Genes in Overlap (k)	k/K	p-value	FDR q-value
HALLMARK_IL2_STAT5_SIGNALING [199]	Genes up-regulated by STAT5 in response to IL2 stimulation.	4		3.67 e-4	1.83 e-2

Gene/geneset overlap matrix

Entrez Gene Id	Gene Symbol	HALLMARK_IL2_STAT5_SIGNALING	Entrez	Ensembl	Gene Description
1028	CDKN1C				cyclin dependent kinase inhibitor 1C [Source:HGNC Symbol;Acc:HGNC:1786]
123	PLIN2				perilipin 2 [Source:HGNC Symbol;Acc:HGNC:248]
214	ALCAM				activated leukocyte cell adhesion molecule [Source:HGNC Symbol;Acc:HGNC:400]
6335	SCN9A				sodium voltage-gated channel alpha subunit 9 [Source:HGNC Symbol;Acc:HGNC:10597]

Figure S3. Analysis of Top Negatively Enriched Genes in GSEA. **A)** Protein-protein interaction (PPI) network of negatively enriched genes with enrichment score < (−1.5). **B)** Overlap of hub genes in PPI network and AF-associated genes found in www.disgenet.org. **C)** Enrichment of Hallmark Pathways in PPI network in Panel A. Lower panel shows overlap matrix of genes and gene sets.

D

Gene Set Name [# Genes (K)]	Description	# Genes in Overlap (k)	k/K	p-value	FDR q-value
REACTOME_NEURONAL_SYSTEM [410]	Neuronal System	10		$1.83\ e^{-9}$	$4.35\ e^{-6}$
KEGG_CELL_ADHESION_MOLECULES_CAMS [133]	Cell adhesion molecules (CAMs)	7		$2.97\ e^{-9}$	$4.35\ e^{-6}$
REACTOME_PROTEIN_PROTEIN_INTERACTIONS_NS_AT_SYNAPSES [87]	Protein-protein interactions at synapses	5		$3.9\ e^{-7}$	$3.8\ e^{-4}$
NABA_MATRISOME [1026]	Ensemble of genes encoding extracellular matrix and extracellular matrix-associated proteins	11		$1.12\ e^{-6}$	$8.19\ e^{-4}$
REACTOME_NEUREXINS_AND_NEUROLIGINS [56]	Neurexins and neuroligins	4		$2.55\ e^{-6}$	$1.49\ e^{-3}$
NABA_MATRISOME_ASSOCIATED [751]	Ensemble of genes encoding ECM-associated proteins including ECM-affilaited proteins, ECM regulators and secreted factors	9		$4.76\ e^{-6}$	$2.32\ e^{-3}$
KEGG_NEUROACTIVE_LIGAND_RECEPTOR_INTERTERACTION [272]	Neuroactive ligand-receptor interaction	6		$6.88\ e^{-6}$	$2.87\ e^{-3}$
REACTOME_CLASS_A_1_RHODOPSIN_LIKE_RECEECEPTORS [331]	Class A/1 (Rhodopsin-like receptors)	6		$2.09\ e^{-5}$	$6.44\ e^{-3}$
REACTOME_DEVELOPMENTAL_BIOLOGY [1143]	Developmental Biology	10		$2.11\ e^{-5}$	$6.44\ e^{-3}$
REACTOME_SIGNALING_BY_GPCR [698]	Signaling by GPCR	8		$2.3\ e^{-5}$	$6.44\ e^{-3}$

Entrez Gene Id	Gene Symbol	REACTOME_NEURONAL_SYSTEM	KEGG_CELL_ADHESION_MOLECULES_CAMS	REACTOME_PROTEIN_PROTEIN_INTERACTIONS_AT_SYNAPSES	NABA_MATRISOME	REACTOME_NEUREXINS_AND_NEUROLIGINS	NABA_MATRISOME_ASSOCIATED	KEGG_NEUROACTIVE_LIGAND_RECEPTOR_INTERACTION	REACTOME_CLASS_A_1_RHODOPSIN_LIKE_RECEPTORS	REACTOME_DEVELOPMENTAL_BIOLOGY	REACTOME_SIGNALING_BY_GPCR	Entrez	Ensembl	Gene Description
9378	NRXN1	■	■	■		■								neurexin 1 [Source:HGNC Symbol;Acc:HGNC:8008]
9369	NRXN3	■	■	■		■								neurexin 3 [Source:HGNC Symbol;Acc:HGNC:8010]
57502	NLGN4X	■	■	■		■								neuroligin 4 X-linked [Source:HGNC Symbol;Acc:HGNC:14287]
6857	SYT1	■		■		■								synaptotagmin 1 [Source:HGNC Symbol;Acc:HGNC:11509]
26050	SLITRK5	■		■										SLIT and NTRK like family member 5 [Source:HGNC Symbol;Acc:HGNC:20295]
2899	GRIK3	■						■						glutamate ionotropic receptor kainate type subunit 3 [Source:HGNC Symbol;Acc:HGNC:4581]
7881	KCNAB1	■												potassium voltage-gated channel subfamily A member regulatory beta subunit 1 [Source:HGNC Symbol;Acc:HGNC:6228]
3737	KCNA2	■												potassium voltage-gated channel subfamily A member 2 [Source:HGNC Symbol;Acc:HGNC:6220]
131096	KCNH8	■												potassium voltage-gated channel subfamily H member 8 [Source:HGNC Symbol;Acc:HGNC:18864]
56666	PANX2	■												pannexin 2 [Source:HGNC Symbol;Acc:HGNC:8600]
3897	L1CAM		■							■				L1 cell adhesion molecule [Source:HGNC Symbol;Acc:HGNC:6470]
214	ALCAM		■							■				activated leukocyte cell adhesion molecule [Source:HGNC Symbol;Acc:HGNC:400]
3118	HLA-DQA2		■											"major histocompatibility complex, class II, DQ alpha 2 [Source:HGNC Symbol;Acc:HGNC:4943]"
3112	HLA-DOB		■											"major histocompatibility complex, class II, DO beta [Source:HGNC Symbol;Acc:HGNC:4937]"
10154	PLXNC1				■		■			■				plexin C1 [Source:HGNC Symbol;Acc:HGNC:9106]
2312	FLG				■		■			■				filaggrin [Source:HGNC Symbol;Acc:HGNC:3748]
7051	TGM1				■		■			■				transglutaminase 1 [Source:HGNC Symbol;Acc:HGNC:11777]
6422	SFRP1				■		■							secreted frizzled related protein 1 [Source:HGNC Symbol;Acc:HGNC:10776]
11197	WIF1				■		■							WNT inhibitory factor 1 [Source:HGNC Symbol;Acc:HGNC:18081]
3699	ITIH3				■		■							inter-alpha-trypsin inhibitor heavy chain 3 [Source:HGNC Symbol;Acc:HGNC:6168]
223117	SEMA3D				■		■							semaphorin 3D [Source:HGNC Symbol;Acc:HGNC:10726]
3083	HGFAC				■		■							HGF activator [Source:HGNC Symbol;Acc:HGNC:4894]
114794	ELFN2				■		■							extracellular leucine rich repeat and fibronectin type III domain containing 2 [Source:HGNC Symbol;Acc:HGNC:29396]
163175	LGI4				■					■				leucine rich repeat LGI family member 4 [Source:HGNC Symbol;Acc:HGNC:18712]
50939	IMPG2				■									interphotoreceptor matrix proteoglycan 2 [Source:HGNC Symbol;Acc:HGNC:18362]
5028	P2RY1							■	■		■			purinergic receptor P2Y1 [Source:HGNC Symbol;Acc:HGNC:8539]
53829	P2RY13							■	■		■			purinergic receptor P2Y13 [Source:HGNC Symbol;Acc:HGNC:4537]
6915	TBXA2R							■	■		■			thromboxane A2 receptor [Source:HGNC Symbol;Acc:HGNC:11608]
186	AGTR2							■	■		■			angiotensin II receptor type 2 [Source:HGNC Symbol;Acc:HGNC:338]
147	ADRA1B							■	■		■			adrenoceptor alpha 1B [Source:HGNC Symbol;Acc:HGNC:278]
3792	KEL								■		■			Kell metallo-endopeptidase (Kell blood group) [Source:HGNC Symbol;Acc:HGNC:6308]
6335	SCN9A									■				sodium voltage-gated channel alpha subunit 9 [Source:HGNC Symbol;Acc:HGNC:10597]
8913	CACNA1G									■				calcium voltage-gated channel subunit alpha1 G [Source:HGNC Symbol;Acc:HGNC:1394]
84059	ADGRV1									■				adhesion G protein-coupled receptor V1 [Source:HGNC Symbol;Acc:HGNC:17416]
1828	DSG1									■				desmoglein 1 [Source:HGNC Symbol;Acc:HGNC:3048]
2780	GNAT2										■			G protein subunit alpha transducin 2 [Source:HGNC Symbol;Acc:HGNC:4394]
126006	PCP2										■			Purkinje cell protein 2 [Source:HGNC Symbol;Acc:HGNC:30209]

Figure S3 Cont. Analysis of Top Positively Enriched Genes in GSEA. **D)** Pathway enrichment analysis of hub genes PPI network in Panel A. Lower panel shows overlap matrix of genes and gene sets.

E

Gene Set Name [# Genes (K)]	Description	# Genes in Overlap (k)	k/K	p-value	FDR q-value
GOCC_INTRINSIC_COMPONENT_OF_PLASMA_MEMBRANE [1730]	The component of the plasma membrane consisting of the gene products and protein complexes having either part of their peptide sequence embedded in the hydrophobic region of the membrane or some other covalently attached group such as a GPI anchor that is similarly embedded in the membrane. [GOC:dos, GOC:mah]	24		$3.17\ e^{-16}$	$3.24\ e^{-12}$
GOBP_NEUROGENESIS [1613]	Generation of cells within the nervous system. [GO_REF:0000021, GOC:cls, GOC:curators, GOC:dgh, GOC:dph, GOC:jid]	22		$1.06\ e^{-14}$	$5.4\ e^{-11}$
GOBP_NEURON_DEVELOPMENT [1109]	The process whose specific outcome is the progression of a neuron over time, from initial commitment of the cell to a specific fate, to the fully functional differentiated cell. [GOC:dph]	19		$1.81\ e^{-14}$	$6.16\ e^{-11}$
GOBP_NEURON_DIFFERENTIATION [1357]	The process in which a relatively unspecialized cell acquires specialized features of a neuron. [GOC:mah]	20		$5.37\ e^{-14}$	$1.37\ e^{-10}$
GOCC_NEURON_PROJECTION [1321]	A prolongation or process extending from a nerve cell, e.g. an axon or dendrite. [GOC:jl, http://www.cogsci.princeton.edu/~wn/]	17		$4.85\ e^{-11}$	$9.94\ e^{-8}$
GOBP_CELL_CELL_SIGNALING [1672]	Any process that mediates the transfer of information from one cell to another. This process includes signal transduction in the receiving cell and, where applicable, release of a ligand and any processes that actively facilitate its transport and presentation to the receiving cell. Examples include signaling via soluble ligands, via cell adhesion molecules and via gap junctions. [GOC:dos, GOC:mah]	18		$2.13\ e^{-10}$	$3.63\ e^{-7}$
GOMF_MOLECULAR_TRANSDUCER_ACTIVITY [1489]	A compound molecular function in which an effector function is controlled by one or more regulatory components. [GOC:dos, GOC:pdt]	17		$3.06\ e^{-10}$	$4.47\ e^{-7}$
GOBP_CELL_PROJECTION_ORGANIZATION [1567]	A process that is carried out at the cellular level which results in the assembly, arrangement of constituent parts, or disassembly of a prolongation or process extending from a cell, e.g. a flagellum or axon. [GOC:jl, GOC:mah, http://www.cogsci.princeton.edu/~wn/]	17		$6.65\ e^{-10}$	$8.5\ e^{-7}$
GOBP_AXON_DEVELOPMENT [507]	The progression of an axon over time. Covers axonogenesis (de novo generation of an axon) and axon regeneration (regrowth), as well as processes pertaining to the progression of the axon over time (fasciculation and defasciculation). [GOC:dph, GOC:pg, GOC:pr]	11		$8.99\ e^{-10}$	$1.02\ e^{-6}$
GOBP_CELL_MORPHOGENESIS [1004]	The developmental process in which the size or shape of a cell is generated and organized. [GOC:clt, GOC:dph, GOC:go_curators, GOC:tb]	14		$1.14\ e^{-9}$	$1.17\ e^{-6}$

Entrez Gene Id	Gene Symbol	GOCC_INTRINSIC_COMPONENT_OF_PLASMA_MEMBRANE	GOBP_NEUROGENESIS	GOBP_NEURON_DEVELOPMENT	GOBP_NEURON_DIFFERENTIATION	GOCC_NEURON_PROJECTION	GOBP_CELL_CELL_SIGNALING	GOMF_MOLECULAR_TRANSDUCER_ACTIVITY	GOBP_CELL_PROJECTION_ORGANIZATION	GOBP_AXON_DEVELOPMENT	GOBP_CELL_MORPHOGENESIS	Entrez	Ensembl	Gene Description
658	BMPR1B	■	■	■	■	■		■	■	■	■			bone morphogenetic protein receptor type 1B [Source:HGNC Symbol;Acc:HGNC:1077]
214	ALCAM	■	■	■	■	■			■	■	■			activated leukocyte cell adhesion molecule [Source:HGNC Symbol;Acc:HGNC:400]
9378	NRXN1	■	■	■	■		■	■	■	■	■			neurexin 1 [Source:HGNC Symbol;Acc:HGNC:8008]
5354	PLP1	■	■	■	■		■		■	■				proteolipid protein 1 [Source:HGNC Symbol;Acc:HGNC:9086]
10154	PLXNC1	■	■	■	■			■	■	■	■			plexin C1 [Source:HGNC Symbol;Acc:HGNC:9106]
9369	NRXN3	■	■	■	■			■	■	■	■			neurexin 3 [Source:HGNC Symbol;Acc:HGNC:8010]
577	ADGRB3	■	■	■	■			■	■		■			adhesion G protein-coupled receptor B3 [Source:HGNC Symbol;Acc:HGNC:945]
223117	SEMA3D	■	■	■	■				■	■	■			semaphorin 3D [Source:HGNC Symbol;Acc:HGNC:10726]
57502	NLGN4X	■	■		■	■	■	■						neuroligin 4 X-linked [Source:HGNC Symbol;Acc:HGNC:14287]
5028	P2RY1	■	■			■	■	■			■			purinergic receptor P2Y1 [Source:HGNC Symbol;Acc:HGNC:8539]
2899	GRIK3	■				■	■	■						glutamate ionotropic receptor kainate type subunit 3 [Source:HGNC Symbol;Acc:HGNC:4581]
3737	KCNA2	■				■	■							potassium voltage-gated channel subfamily A member 2 [Source:HGNC Symbol;Acc:HGNC:6220]
8913	CACNA1G	■				■	■							calcium voltage-gated channel subunit alpha1 G [Source:HGNC Symbol;Acc:HGNC:1394]
1259	CNGA1	■				■								cyclic nucleotide gated channel subunit alpha 1 [Source:HGNC Symbol;Acc:HGNC:2148]
6335	SCN9A	■				■								sodium voltage-gated channel alpha subunit 9 [Source:HGNC Symbol;Acc:HGNC:10597]
7881	KCNAB1	■				■								potassium voltage-gated channel subfamily A member regulatory beta subunit 1 [Source:HGNC Symbol;Acc:HGNC:6228]
147	ADRA1B	■					■	■						adrenoceptor alpha 1B [Source:HGNC Symbol;Acc:HGNC:278]
186	AGTR2	■					■	■						angiotensin II receptor type 2 [Source:HGNC Symbol;Acc:HGNC:338]
56130	PCDHB6	■					■							protocadherin beta 6 [Source:HGNC Symbol;Acc:HGNC:8691]
6915	TBXA2R	■						■						thromboxane A2 receptor [Source:HGNC Symbol;Acc:HGNC:11608]
3118	HLA-DQA2	■						■						"major histocompatibility complex, class II, DQ alpha 2 [Source:HGNC Symbol;Acc:HGNC:4943]"
145581	LRFN5	■												leucine rich repeat and fibronectin type III domain containing 5 [Source:HGNC Symbol;Acc:HGNC:20360]
131096	KCNH8	■												potassium voltage-gated channel subfamily H member 8 [Source:HGNC Symbol;Acc:HGNC:18864]
6005	RHAG	■												Rh associated glycoprotein [Source:HGNC Symbol;Acc:HGNC:10006]
6857	SYT1		■	■	■	■	■		■		■			synaptotagmin 1 [Source:HGNC Symbol;Acc:HGNC:11509]
23418	CRB1		■	■	■	■	■				■			crumbs cell polarity complex component 1 [Source:HGNC Symbol;Acc:HGNC:2343]
3897	L1CAM		■	■	■	■		■	■	■	■			L1 cell adhesion molecule [Source:HGNC Symbol;Acc:HGNC:6470]
84059	ADGRV1		■	■	■	■		■	■					adhesion G protein-coupled receptor V1 [Source:HGNC Symbol;Acc:HGNC:17416]
94137	RP1L1		■	■	■	■			■					RP1 like 1 [Source:HGNC Symbol;Acc:HGNC:15946]
9241	NOG		■	■	■		■		■	■	■			noggin [Source:HGNC Symbol;Acc:HGNC:7866]
26050	SLITRK5		■	■	■		■		■	■	■			SLIT and NTRK like family member 5 [Source:HGNC Symbol;Acc:HGNC:20295]
6422	SFRP1		■	■	■		■		■					secreted frizzled related protein 1 [Source:HGNC Symbol;Acc:HGNC:10776]
3792	KEL		■	■	■				■	■	■			Kell metallo-endopeptidase (Kell blood group) [Source:HGNC Symbol;Acc:HGNC:6308]
2824	GPM6B		■	■	■				■					glycoprotein M6B [Source:HGNC Symbol;Acc:HGNC:4461]
163175	LGI4		■	■	■									leucine rich repeat LGI family member 4 [Source:HGNC Symbol;Acc:HGNC:18712]
745	MYRF		■											myelin regulatory factor [Source:HGNC Symbol;Acc:HGNC:1181]
2780	GNAT2					■	■	■						G protein subunit alpha transducin 2 [Source:HGNC Symbol;Acc:HGNC:4394]
5874	RAB27B					■								"RAB27B, member RAS oncogene family [Source:HGNC Symbol;Acc:HGNC:9767]"
11197	WIF1						■							WNT inhibitory factor 1 [Source:HGNC Symbol;Acc:HGNC:18081]
56666	PANX2						■							pannexin 2 [Source:HGNC Symbol;Acc:HGNC:8600]
3112	HLA-DOB							■						"major histocompatibility complex, class II, DO beta [Source:HGNC Symbol;Acc:HGNC:4937]"
53829	P2RY13							■						purinergic receptor P2Y13 [Source:HGNC Symbol;Acc:HGNC:4537]

Figure S3. Analysis of Top Negatively Enriched Genes in GSEA. **E)** GO terms of negatively enriched genes in PPI network and gene/gene-set overlap matrix
